# Global, regional, and national burdens of rheumatoid arthritis among people aged 60 years and older from 1990 to 2021: a trend analysis for the Global Burden of Disease Study 2021

**DOI:** 10.3389/fpubh.2025.1527680

**Published:** 2025-05-26

**Authors:** Qin-Yi Su, Liu Yang, Xiang-Yu Qi, Meng-Yuan Wang, Jing-Wen Cheng, Han Niu, Sheng-Xiao Zhang

**Affiliations:** ^1^Department of Rheumatology, The Second Hospital of Shanxi Medical University, Taiyuan, China; ^2^Shanxi Provincial Key Laboratory of Rheumatism Immune Microecology, Taiyuan, China; ^3^Key Laboratory of Cellular Physiology at Shanxi Medical University, Ministry of Education, Taiyuan, China; ^4^Department of Gynaecology and Obstetrics, Shanxi Bethune Hospital, Third Hospital of Shanxi Medical University, Taiyuan, China

**Keywords:** rheumatoid arthritis, Global Burden of Disease (GBD), incidence, disability-adjusted life-years (DALYs), trend

## Abstract

**Background:**

Rheumatoid arthritis (RA) poses a significant global health burden, especially among individuals aged 60 and older. This study analyzes RA burden trends using 2021 Global Burden of Disease (GBD) data.

**Methods:**

Utilizing the GBD 2021 data, we extracted detailed metric on RA incidence, prevalence, mortality, and disability-adjusted life years (DALYs) specifically for those aged 60 and above. Linear regression analysis was applied to calculate the overall average annual percentage change (AAPC) spanning from 1990 to 2021. Furthermore, Joinpoint regression was employed to identify the years with the most significant changes in global trends. A nuanced stratified analysis was also performed, examining global trends across age groups, genders, and sociodemographic indices (SDI).

**Results:**

From 1990 to 2021, both the age-standardized incidence rate (ASIR) and age-standardized prevalence rate (ASPR) of RA in people aged 60 and above worldwide has increased ASIR rose from 24.87 per 100,000 to 30.32 per 100,000 (95% uncertainty interval [UI] 19.83–42.38), while ASPR climbed from 635.51 per 100,000 to 726.91 per 100,000 (95% UI 634.05–834.80). Conversely, the age-standardized death rate (ASDR) and age-standardized DALYs rate declined, with ASDR decreasing from 4.18 per 100,000 to 3.20 per 100,000 (95% UI 2.58–3.72), and DALYs rate dropping from 150.83 per 100,000 to 143.20 per 100,000 (95% UI 113.39–178.43). Regionally, ASIR surged in North Africa and Middle East, Southeast Asia, and Andean Latin America, with ASPR peaking in Andean Latin America. Central Europe saw ASDR decline, while Central Asia’s DALYs rate rose. Nationally, Vietnam’s ASIR soared, and Ireland topped ASIR, ASPR, and DALYs rates. In 2021, for the global population aged 60 and above with RA: ASIR peaked at 65–69 years, ASPR peaked at 75–79 years; ASDR rose with age, and the age-standardized DALYs rate peaked at 85–89 years. Women bore a heavier RA burden. A non-linear SDI-DALYs relationship was noted, with Honduras and Mexico having high DALYs rates.

**Conclusion:**

From 1990 to 2021, the global ASIR and ASPR of RA increased among the older adult, while ASDR and age-standardized DALYs rate declined. Despite improvements, RA remains a public health priority, necessitating enhanced early diagnosis, treatment, and awareness, particularly among older adult women.

## 1 Introduction

Rheumatoid arthritis (RA) is a prevalent autoimmune disease characterized by chronic inflammation of joint tissues (1, 2). It can affect any synovial joint, with initial symptoms often manifesting as swelling, pain, and deformation in the small joints of hands and feet, potentially progressing to larger joints (3). RA impacts individuals of all ages, including males, females, and children, exhibiting significant regional variations in incidence and prevalence worldwide (4). Research indicates that the incidence of RA increases with age, peaking between 60 and 70 years. In the absence of adequate treatment, the disease can lead to progressive joint damage, deformity, chronic pain, long-term disability, and premature mortality (5). Additionally, RA imposes substantial socioeconomic burdens due to ongoing medical needs and a reduced quality of life (6).

Previous studies have reported the global burden of RA at regional or national levels using World Health Organization (WHO) databases or older Global Burden of Disease (GBD) versions (7–11). In addition to assessing the overall burden of RA, previous research has primarily focused on specific patient groups, such as women of reproductive age, children and adolescents, and the working-age population (6, 12–15). While these studies have provided valuable insights into the epidemiology and risk factors associated with RA, a comprehensive analysis specifically addressing the older adult population remains absent. In recent years, RA prevalence and associated health burdens have risen in many countries, aligning with population aging trends (16). Given the rapid global aging of the population and the unique clinical and socioeconomic challenges posed by RA in older adults, it is crucial to investigate its burden among individuals aged 60 years and older. To further investigate the correlation between RA and regional aging populations, we present the first study utilizing GBD 2021 to assess the global, regional, and national burdens of RA among individuals aged 60 years and older.

Our research leverages data from the GBD 2021 study, enabling cross-geographical, temporal, age, and gender comparisons. By integrating data from multiple sources, including life registration systems, censuses, health surveys, and epidemiological studies, and employing sophisticated statistical models, we aim to provide valuable insights into long-term trends of RA burden associated with population aging. This study aims to inform healthcare policies, resource allocation, and intervention strategies to effectively address the significant impact of RA, particularly within the context of a globally aging population.

## 2 Methods

### 2.1 Study population and data collection

This study is based on data from the “Global Burden of Diseases, Injuries, and Risk Factors Study 2021” (GBD 2021) released by the Institute for Health Metrics and Evaluation (IHME). All data are open-source and publicly accessible. GBD 2021 represents the most comprehensive and detailed assessment of global diseases, injuries, and risk factors to date, covering 204 countries and regions and estimating the burden of 369 diseases and injuries, including RA, from 1990 to 2021. The International Classification of Diseases (ICD)-9 codes 714–714.3, 714.8–714.9 and ICD-10 codes M05-M06.9, M08.0-M08.8 form the basis for the case definition of RA in the GBD (17).

The study provides estimates of RA incidence, prevalence, mortality, and associated disability across these 204 countries and regions, stratified by year, age, and gender. Data are presented at three levels: global, regional, and national. Regional and national classifications in the GBD study follow the framework established by the IHME. Regions are groupings of countries based on epidemiological similarity and geographic proximity. For example, the ‘Andean Latin America’ region includes countries such as Bolivia, Ecuador, and Peru. The national level refers to the direct classification of individual countries and territories, encompassing a total of 204 distinct entities.

In this study, the population of interest is individuals aged 60 years and older. We utilized the GBD database’s age stratification, which organizes data into five-year increments. By amalgamating eight specific age brackets, we conducted a thorough analysis of the burden of RA among individuals aged 60 and older. The age groups considered include: 60–64, 65–69, 70–74, 75–79, 80–84, 85–89, 90–94, and those aged 95 and above. The study focuses on trends in four key indicators: incidence, prevalence, mortality, and disability-adjusted life years (DALYs). DALYs are a measure of disease burden that combines the number of years of life lost due to premature death and the number of years lived with disability. DALYs for RA in each group are calculated using relevant data from GBD 2021, and further analysis of trends is conducted. To gain a more detailed understanding of the 60 + age group and their health outcomes, GBD 2021 also calculates the Socio-demographic Index (SDI) for each country. The SDI is a composite measure of the social and economic conditions that affect health outcomes in each region. It is formulated based on indicators such as education level, income per capita, and fertility rate. The SDI ranges from 0 to 1, with higher values indicating a higher level of socio-economic development. The SDI is divided into five quintiles: low, low-middle, middle, high-middle, and high.

### 2.2 Statistical analyses

The first objective is to examine the global trends in incidence, prevalence, mortality, and DALYs for the 60+ age group. We calculated age-standardized rates for the entire age group and used linear regression to compute the average annual percent change (AAPC) from 1990 to 2021. The AAPC is a summary measure of trend over a prespecified fixed interval and is calculated as the weighted average of the annual percent change (APC), allowing us to describe the average APC over multiple years with a single number. The APC is calculated as the geometric weighted average of the annual percent changes derived from regression analysis. The AAPC value represents the percentage change per year (increase, decrease, or no change). For example, an AAPC of 0.1 indicates an annual growth rate of 0.1%. Trends in incidence, prevalence, mortality, and DALYs are reflected in the AAPC values and their 95% confidence intervals.

The second objective is to identify the years with the greatest changes in trends for the aforementioned indicators. We used joinpoint regression analysis to determine the trends in age-standardized prevalence over time. The simplest model fits several different line segments on a logarithmic scale, known as joinpoints, which is tested using the Monte Carlo permutation method. The final model is selected using the weighted Bayesian Information Criterion method and recommendations provided by the Joinpoint software.

The third objective is to stratify the global trends by age group, gender, and SDI. Global trend analysis and stratification by these factors was conducted using the same method for calculating AAPC as described above. All statistical analyses were performed using GraphPad Prism (8.0.2), RStudio software (R4.3.3), and the Joinpoint Regression Program (4.9.1.0).

## 3 Results

### 3.1 Global level

**Figure 1:**
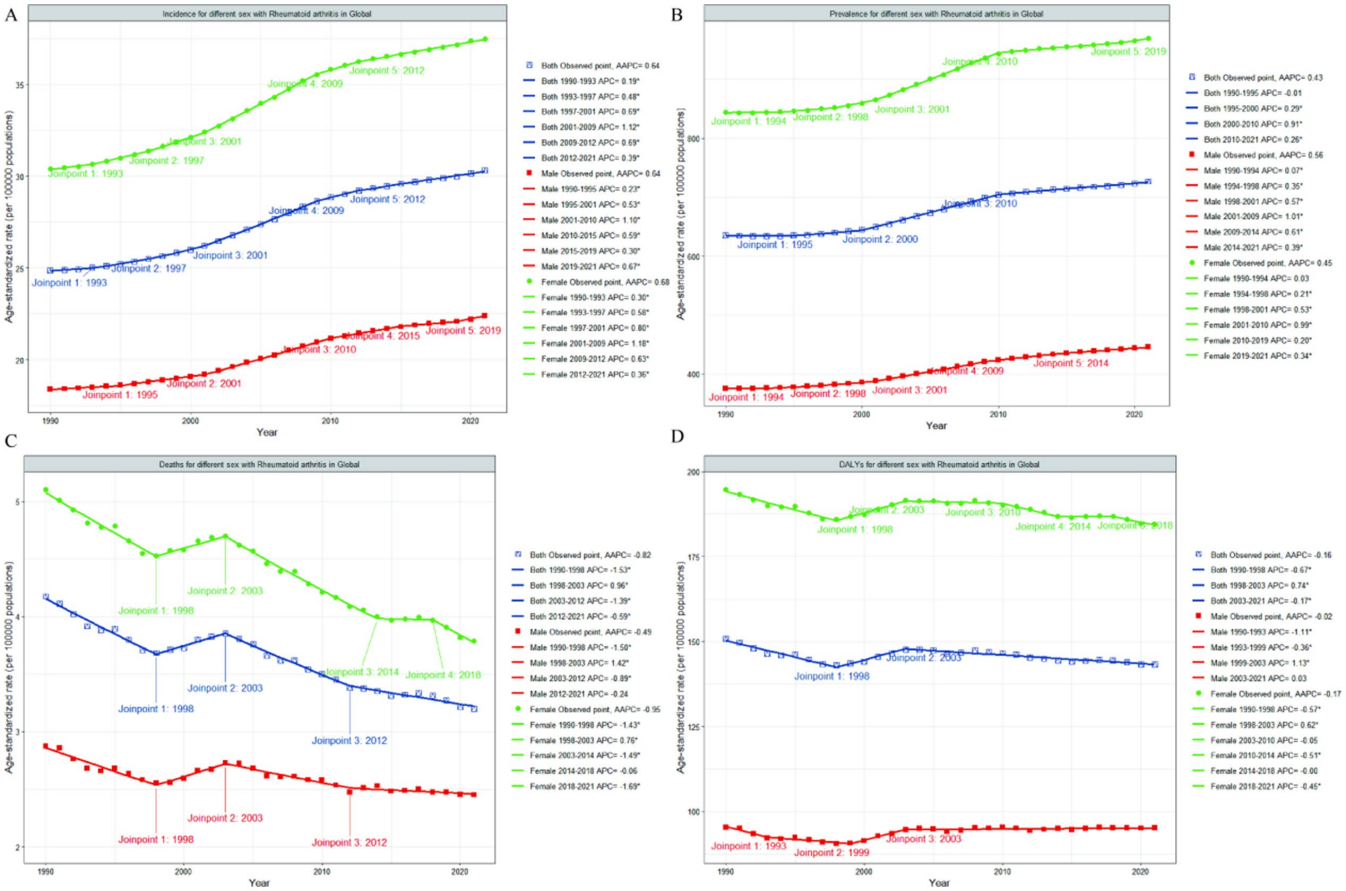
Joinpoint regression analysis of global RA ASIR **(A)**, ASPR **(B)**, ASDR **(C)** and age-standardized DALYs rate **(D)** among people aged 60 years and older from 1990 to 2021. That the APC is significantly different from zero at the alpha = 0.05 level. RA, rheumatoid arthritis; ASIR, age-standardized incidence rate; ASPR, age-standardized prevalence rate; ASDR, age-standardized death rate; DALYs, disability-adjusted life-years; APC, annual percentage change; AAPC, average annual percentage change.

Globally, the age-standardized incidence rate (ASIR) of RA among individuals aged 60 years and above showed an upward trend from 1990 to 2021, with an AAPC of 0.64 (95% CI 0.60 to 0.68); [from 24.87 per 100,000 population (95% UI 15.88–35.31) in 1990 to 30.32 per 100,000 population (19.83–42.38) in 2021] (Table 1). Joinpoint regression analysis revealed substantial changes in the ASIR of RA in 1993, 1997, 2001, 2009, and 2012, with the most significant increase observed from 2001 to 2009 (Figure 1A). The age-standardized prevalence rate (ASPR) of RA among those aged 60 and above also generally increased during 1990–2021, with an AAPC of 0.43 (95% CI 0.42 to 0.45; from 635.51 per 100,000 population [95% UI 551.54–735.47] in 1990 to 726.91 per 100,000 population [634.05–834.80] in 2021), although a slight decrease was noted from 1990 to 1995 (Table 2 and Figure 1B). The age-standardized death rate (ASDR) of RA among individuals aged 60 and above declined across most periods except from 1998 to 2003. Overall, the ASDR of RA in 2021 (3.20 per 100,000 population [95% UI 2.58–3.72]) was lower than that in 1990 (4.18 per 100,000 population [95% UI 3.60–4.74]); AAPC -0.82 [95% CI -0.96 to −0.68] (Table 3 and Figure 1C). The age-standardized DALYs rate for RA among those aged 60 and above also decreased from 1990 to 2021, but it rose slightly between 1998 and 2003. In total, the age-standardized DALYs rate for RA in 2021 (143.20 per 100,000 population [95% UI 113.39–178.43]) was lower than that in 1990 (150.83 per 100,000 population [95% UI 123.59–182.01]); AAPC −0.16 [95% CI -0.21 to −0.10] (Table 4 and Figure 1D).

**Table 1:** The ASIR and AAPCs of RA among people aged 60 years and older from 1990 to 2021.

Factors	Incidence per 100,000 population (95% UI) in 1990	Incidence per 100,000 population (95% UI) in 2021	AAPCs (95% CI), 1990 to 2021	*p*-value^*^
Global	24.87(15.88,35.31)	30.32(19.83,42.38)	0.64(0.60,0.68)	<0.001
Sex				
Male	18.41(11.81,26.00)	22.41(14.76,31.02)	0.64(0.61,0.66)	<0.001
Female	30.39(19.40,43.21)	37.49(24.42,52.58)	0.68(0.65,0.71)	<0.001
Age group				
60 to 64	25.98(16.18,38.39)	30.76(19.65,44.27)	0.54(0.48,0.61)	<0.001
65 to 69	28.29(17.74,39.25)	34.73(22.56,47.51)	0.65(0.58,0.73)	<0.001
70 to 74	27.26(16.61,40.43)	34.32(21.34,49.95)	0.74(0.69,0.79)	<0.001
75 to 79	23.97(17.21,31.09)	30.20(22.16,39.04)	0.75(0.72,0.77)	<0.001
80 to 84	19.69(13.32,26.70)	23.25(15.67,31.92)	0.53(0.49,0.57)	<0.001
85 to 89	13.92(9.44,18.87)	16.34(11.06,22.34)	0.51(0.45,0.56)	<0.001
90 to 94	8.02(5.42,10.88)	9.14(6.17,12.45)	0.43(0.39,0.47)	<0.001
95 plus	1.95(1.31,2.64)	2.22(1.50,3.02)	0.41(0.37,0.46)	<0.001
Sociodemographic index				
High	40.73(27.06,56.52)	43.61(29.71,59.75)	0.22(0.20,0.24)	<0.001
High-middle	15.78(9.69,23.33)	22.29(14.28,31.76)	1.12(1.10,1.15)	<0.001
Middle	19.90(12.00,29.35)	27.16(17.08,39.11)	1.01(0.99,1.04)	<0.001
Low-middle	23.36(14.92,33.17)	32.66(21.14,45.79)	1.09(1.05,1.13)	<0.001
Low	15.51(9.91,22.12)	21.42(13.79,30.05)	1.05(1.03,1.07)	<0.001
Region				
Andean Latin America	22.33(15.92,29.77)	32.47(21.75,45.07)	1.22(1.17,1.27)	<0.001
Australasia	66.13(44.59,90.44)	74.56(50.92,102.49)	0.39(0.36,0.41)	<0.001
Caribbean	15.21(11.09,20.06)	18.84(13.66,25.03)	0.68(0.64,0.73)	<0.001
Central Asia	6.24(3.79,9.33)	8.35(5.02,12.35)	0.94(0.90,0.98)	<0.001
Central Europe	7.34(4.00,11.48)	10.26(5.81,15.74)	1.09(1.06,1.12)	<0.001
Central Latin America	34.45(21.84,49.45)	33.98(21.81,48.51)	−0.06(−0.10,−0.01)	0.011
Central Sub-Saharan Africa	10.25(6.49,14.53)	11.54(7.10,16.60)	0.38(0.34,0.43)	<0.001
East Asia	22.68(13.21,34.51)	29.45(18.35,42.89)	0.86(0.83,0.88)	<0.001
Eastern Europe	2.97(1.46,4.95)	3.68(1.88,6.03)	0.69(0.63,0.75)	<0.001
Eastern Sub-Saharan Africa	10.96(6.92,15.71)	12.43(7.84,17.71)	0.40(0.38,0.42)	<0.001
High-income Asia Pacific	44.38(27.15,65.80)	38.47(24.59,54.76)	−0.46(−0.50,−0.41)	<0.001
High-income North America	40.11(26.60,55.33)	48.39(32.84,65.77)	0.62(0.60,0.64)	<0.001
North Africa and Middle East	3.12(1.66,4.94)	4.86(2.66,7.57)	1.44(1.40,1.47)	<0.001
Oceania	2.46(1.34,3.84)	2.83(1.60,4.32)	0.46(0.41,0.52)	<0.001
South Asia	34.41(21.98,49.10)	49.11(31.50,69.26)	1.16(1.13,1.19)	<0.001
Southeast Asia	5.94(3.54,8.74)	8.64(5.27,12.52)	1.23(1.18,1.28)	<0.001
Southern Latin America	19.23(12.87,26.62)	26.38(16.96,37.86)	1.02(1.00,1.04)	<0.001
Southern Sub-Saharan Africa	16.89(10.12,25.42)	17.84(10.76,26.64)	0.19(0.15,0.23)	<0.001
Tropical Latin America	5.22(2.74,8.44)	5.58(3.04,8.88)	0.25(0.22,0.27)	<0.001
Western Europe	41.50(28.19,57.00)	47.03(32.48,63.77)	0.40(0.37,0.43)	<0.001
Western Sub-Saharan Africa	5.04(3.00,7.48)	6.03(3.67,8.76)	0.59(0.56,0.62)	<0.001

ASIR, age-standardized incidence rate; UI, uncertainty interval; AAPCs, average annual percentage changes; CI, confidence interval. ^*^The *p*-value was determined by joinpoint regression analysis.

**Table 2:** The ASPR and AAPCs of RA among people aged 60 years and older from 1990 to 2021.

Factors	Prevalence per 100,000 population (95% UI) in 1990	Prevalence per 100,000 population (95% UI) in 2021	AAPCs (95% CI), 1990 to 2021	*P*-value^*^
Global	635.51(551.54,735.47)	726.91(634.05,834.80)	0.43(0.42,0.45)	<0.001
Sex				
Male	375.29(319.38,442.89)	445.74(383.91,519.76)	0.56(0.54,0.58)	<0.001
Female	844.21(736.41,969.79)	969.92(849.90,1108.45)	0.45(0.43,0.47)	<0.001
Age group				
60 to 64	541.76(464.36,637.84)	608.69(524.53,707.78)	0.37(0.31,0.43)	<0.001
65 to 69	649.23(555.78,762.28)	724.29(624.53,842.51)	0.35(0.28,0.41)	<0.001
70 to 74	698.32(605.94,808.28)	806.12(703.07,926.64)	0.46(0.42,0.50)	<0.001
75 to 79	711.26(626.32,809.79)	836.22(736.28,947.88)	0.52(0.49,0.56)	<0.001
80 to 84	696.58(618.52,779.64)	809.19(721.92,906.32)	0.48(0.45,0.52)	<0.001
85 to 89	643.72(576.17,717.37)	765.68(609.27,757.59)	0.56(0.53,0.59)	<0.001
90 to 94	568.47(508.45,637.21)	678.53(609.27,757.59)	0.58(0.54,0.61)	<0.001
95 plus	450.76(396.67,512.86)	565.25(499.87,643.11)	0.72(0.68,0.77)	<0.001
Sociodemographic index				
High	997.50(873.17,1146.97)	1031.16(911.68,1171.86)	0.11(0.07,0.16)	<0.001
High-middle	513.64(446.17,591.35)	643.97(563.55,735.07)	0.73(0.72,0.75)	<0.001
Middle	514.83(438.93,603.54)	654.36(563.97,761.69)	0.78(0.77,0.79)	<0.001
Low-middle	409.90(345.83,487.95)	617.38(527.93,725.77)	1.33(1.31,1.36)	<0.001
Low	290.24(246.67,341.93)	408.62(350.71,478.39)	1.11(1.08,1.14)	<0.001
Region				
Andean Latin America	604.01(539.57,674.17)	1058.6(949.15,1177.15)	1.83(1.78,1.88)	<0.001
Australasia	1388.18(1216.86,1576.19)	1478.59(1297.40,1680.18)	0.21(0.18,0.24)	<0.001
Caribbean	333.34(293.33,378.94)	465.28(412.68,524.08)	1.08(1.07,1.09)	<0.001
Central Asia	292.04(259.21,329.34)	403.41(362.49,448.67)	1.04(0.97,1.12)	<0.001
Central Europe	477.65(422.29,540.40)	581.56(516.02,653.65)	0.64(0.62,0.65)	<0.001
Central Latin America	887.51(768.90,1025.01)	1047.26(926.70,1186.83)	0.54(0.51,0.56)	<0.001
Central Sub-Saharan Africa	266.99(234.21,304.00)	333.00(292.63,379.49)	0.71(0.69,0.73)	<0.001
East Asia	643.13(545.57,759.91)	745.47(639.68,870.34)	0.48(0.47,0.49)	<0.001
Eastern Europe	326.61(288.91,369.14)	391.26(349.68,437.29)	0.59(0.57,0.61)	<0.001
Eastern Sub-Saharan Africa	250.07(214.96,292.27)	287.87(249.70,331.75)	0.45(0.42,0.48)	<0.001
High-income Asia Pacific	1202.75(1015.38,1428.80)	1000.26(862.00,1163.70)	−0.58(−0.64,−0.52)	<0.001
High-income North America	936.68(827.40,1063.96)	1097.02(975.70,1234.83)	0.52(0.48,0.55)	<0.001
North Africa and Middle East	161.36(140.98,185.45)	271.52(240.71,306.37)	1.70(1.67,1.73)	<0.001
Oceania	92.06(77.22,109.87)	110.11(94.16,129.19)	0.58(0.54,0.62)	<0.001
South Asia	541.58(450.10,654.74)	827.91(697.26,986.35)	1.38(1.36,1.41)	<0.001
Southeast Asia	158.03(133.83,186.44)	229.75(197.44,266.90)	1.21(1.19,1.24)	<0.001
Southern Latin America	479.79(422.01,546.92)	802.42(718.10,897.08)	1.67(1.66,1.68)	<0.001
Southern Sub-Saharan Africa	624.8(546.76,715.58)	612.03(536.77,701.59)	−0.07(−0.09,−0.05)	<0.001
Tropical Latin America	308.35(265.27,357.06)	315.72(275.28,362.79)	0.06(0.01,0.11)	0.011
Western Europe	955.19(834.75,1098.49)	1029.85(902.95,1172.25)	0.24(0.21,0.27)	<0.001
Western Sub-Saharan Africa	134.12(111.81,159.80)	166.04(140.90,195.17)	0.70(0.67,0.73)	<0.001

ASPR, age-standardized prevalence rate. UI, uncertainty interval. AAPCs, average annual percentage changes. CI, confidence interval. ^*^The *P*-value was determined by joinpoint regression analysis.

**Table 3:** The ASDR and AAPCs of RA among people aged 60 years and older from 1990 to 2021.

Factors	Death per 100,000 population (95% UI) in 1990	Death per 100,000 population (95% UI) in 2021	AAPCs (95% CI), 1990 to 2021	*P*-value^*^
Global	4.18(3.60,4.74)	3.20(2.58,3.72)	−0.82(−0.96,−0.68)	<0.001
Sex				
Male	2.87(2.12,3.39)	2.45(1.58,2.98)	−0.49(−0.66,−0.31)	<0.001
Female	5.10(4.41,5.86)	3.79(3.07,4.55)	−0.95(−1.12,−0.78)	<0.001
Age group				
60 to 64	1.38(1.23,1.56)	0.87(0.74,1.01)	−1.47(−1.63,−1.30)	<0.001
65 to 69	2.44(2.16,2.76)	1.63(1.36,1.91)	−1.23(−1.61,−0.85)	<0.001
70 to 74	3.98(3.49,4.57)	2.85(2.35,3.28)	−1.06(−1.26,−0.86)	<0.001
75 to 79	6.23(5.53,6.93)	4.85(4.06,5.49)	−0.82(−1.11,−0.52)	<0.001
80 to 84	7.96(6.75,9.00)	6.78(5.32,7.91)	−0.51(−0.76,−0.25)	<0.001
85 to 89	12.25(10.23,14.09)	10.16(7.85,12.13)	−0.6(−0.81,−0.39)	<0.001
90 to 94	14.86(11.84,17.12)	12.74(9.59,14.80)	−0.42(−0.64,−0.21)	<0.001
95 plus	16.23(12.50,18.74)	15.00(10.79,17.37)	−0.27(−0.73,0.19)	0.245
Sociodemographic index				
High	5.28(4.80,5.57)	2.79(2.38,3.04)	−2.07(−2.50,−1.63)	<0.001
High-middle	2.64(2.31,3.00)	2.50(2.01,2.93)	−0.23(−0.65,0.20)	<0.001
Middle	4.25(3.42,4.98)	3.47(2.62,4.05)	−0.68(−1.02,−0.33)	<0.001
Low-middle	4.89(3.28,7.14)	4.86(3.45,6.96)	0.08(−0.26,0.41)	<0.001
Low	2.65(1.64,4.74)	2.69(1.74,4.59)	0.05(−0.69,0.80)	<0.001
Region				
Andean Latin America	6.63(4.91,8.39)	4.16(3.16,5.39)	−1.56(−0.24,−0.71)	<0.001
Australasia	6.89(5.88,7.95)	3.60(2.90,4.32)	−2.06(−2.34,−1.79)	<0.001
Caribbean	3.78(3.14,4.48)	3.07(2.48,3.75)	−0.67(−1.07,−0.28)	0.001
Central Asia	0.21(0.16,0.33)	1.17(0.99,1.38)	5.26(1.84,8.81)	0.002
Central Europe	3.18(2.98,3.37)	1.47(1.31,1.62)	−2.58(−3.21,−1.94)	<0.001
Central Latin America	10.22(9.47,10.84)	6.16(5.31,6.99)	−1.63(−2.01,−1.26)	<0.001
Central Sub-Saharan Africa	0.43(0.12,3.97)	0.30(0.07,3.30)	−1.23(−1.31,−1.15)	<0.001
East Asia	4.51(3.57,5.69)	3.79(2.61,4.70)	−0.58(−0.91,−0.25)	0.001
Eastern Europe	1.73(1.61,1.82)	2.19(11.97,2.41)	0.77(−0.11,1.66)	0.087
Eastern Sub-Saharan Africa	0.28(0.09,2.24)	0.17(0.05,1.69)	−1.62(−1.72,−1.52)	<0.001
High-income Asia Pacific	6.41(5.68,6.97)	3.11(2.53,3.50)	−2.25(−2.96,−1.54)	<0.001
High-income North America	3.61(3.26,3.84)	2.59(2.21,2.87)	−1.19495059356	<0.001
North Africa and Middle East	1.04(0.63,1.59)	0.81(0.56,1.10)	−0.74(−0.88,−0.60)	<0.001
Oceania	0.01(0.00,0.02)	0.00(0.00,0.01)	−2.13(−2.61,−1.65)	<0.001
South Asia	6.66(4.27,10.01)	5.92(4.17,8.91)	−0.34(−0.90,0.22)	0.233
Southeast Asia	1.51(0.89,2.00)	1.45(0.90,1.84)	−0.13(−0.19,−0.06)	<0.001
Southern Latin America	3.24(2.81,3.67)	2.85(2.39,3.31)	−0.47(−1.54,0.61)	0.391
Southern Sub-Saharan Africa	5.21(3.56,6.72)	4.44(3.50,5.69)	−0.49(−0.33,−0.93)	0.08
Tropical Latin America	1.65(1.48,1.81)	1.55(1.34,1.72)	−0.33(−0.93,0.28)	0.287
Western Europe	5.33(4.83,5.69)	2.58(2.20,2.83)	−2.37(−2.83,−1.90)	<0.001
Western Sub-Saharan Africa	0.06(0.03,0.11)	0.07(0.03,0.15)	0.45(0.20,0.70)	<0.001

ASDR, age-standardized death rate. UI, uncertainty interval. AAPCs, average annual percentage changes. CI, confidence interval. ^*^The *P*-value was determined by joinpoint regression analysis.

**Table 4:** The age-standardized DALYs rate and AAPCs of RA among people aged 60 years and older from 1990 to 2021.

Factors	DALYs per 100,000 population (95% UI) in 1990	DALYs per 100,000 population (95% UI) in 2021	AAPCs (95% CI), 1990 to 2021	*P*-value^*^
Global	150.83(123.59,182.01)	143.20(113.39,178.43)	−0.16(−0.21,−0.10)	<0.001
Sex				
Male	95.32(75.81,117.66)	95.15(72.91,119.02)	−0.02(−0.12,0.08)	0.752
Female	194.78(159.69,235.76)	184.55(143.82,231.37)	−0.17(−0.27,−0.07)	0.001
Age group				
60 to 64	110.59(87.30,138.87)	104.32(78.56,137.00)	−0.19(−0.27,−0.11)	<0.001
65 to 69	142.67(115.27,175.14)	132.07(103.55,168.99)	−0.26(−0.42,−0.10)	0.002
70 to 74	167.51(138.18,201.47)	157.87(125.64,196.31)	−0.20(−0.35,−0.06)	0.006
75 to 79	187.16(156.41,220.97)	179.99(146.90,216.25)	−0.11(−0.28,0.05)	0.172
80 to 84	183.28(153.94,212.81)	181.51(147.05,215.22)	−0.04(−0.19,0.11)	0.58
85 to 89	197.31(169.22,227.46)	190.03(156.92,223.54)	−0.10(−0.19,0.00)	0.044
90 to 94	193.43(164.12,221.31)	187.17(154.74,216.37)	−0.06(−0.24,0.11)	0.46
95 plus	182.63(150.83,209.15)	184.05(151.04,213.37)	0(−0.34,0.33)	0.983
Sociodemographic index				
High	217.24(177.05,264.73)	174.42(133.23,222.92)	−0.71(−0.84,−0.58)	<0.001
High-middle	112.78(91.55,139.05)	122.48(95.47,154.97)	0.26(0.11,0.41)	0.001
Middle	133.15(108.63,163.16)	137.25(108.36,170.43)	0.09(−0.06,0.24)	0.237
Low-middle	134.24(100.83,172.58)	155.99(121.12,198.88)	0.53(0.29,0.78)	<0.001
Low	80.92(59.69,115.10)	95.26(72.00,128.61)	0.55(0.22,0.87)	0.001
Region				
Andean Latin America	165.87(128.58,207.43)	194.70(147.21,248.91)	0.51(0.00,1.03)	0.052
Australasia	292.23(232.38,363.44)	242.95(181.33,315.36)	−0.65(−0.74,−0.56)	<0.001
Caribbean	93.95(75.80,114.06)	102.90(81.92,127.56)	0.29(0.23,0.34)	<0.001
Central Asia	40.53(28.01,56.06)	69.62(52.74,90.72)	1.68(0.90,2.46)	<0.001
Central Europe	121.75(103.01,144.37)	100.54(77.56,128.43)	−0.66(−0.90,−0.42)	<0.001
Central Latin America	264.95(227.19,308.29)	235.36(192.20,286.77)	−0.37(−0.55,−0.19)	<0.001
Central Sub-Saharan Africa	41.13(26.09,103.43)	46.94(30.20,97.84)	0.43(0.35,0.50)	<0.001
East Asia	156.01(125.04,196.35)	152.94(117.55,193.82)	−0.07(−0.22,0.08)	0.375
Eastern Europe	78.93(65.41,95.13)	93.01(77.68,112.06)	0.55(0.14,0.95)	0.008
Eastern Sub-Saharan Africa	36.20(24.39,68.36)	39.06(26.59,64.42)	0.24(0.22,0.26)	<0.001
High-income Asia Pacific	263.75(212.28,325.83)	176.79(134.65,227.33)	−1.24(−1.51,−0.97)	<0.001
High-income North America	182.89(145.59,226.43)	178.24(135.91,226.54)	−0.08(−0.22,0.07)	0.318
North Africa and Middle East	39.23(29.07,52.47)	48.62(36.37,63.54)	0.71(0.63,0.78)	<0.001
Oceania	12.55(8.00,18.35)	14.55(9.04,21.70)	0.47(0.41,0.53)	<0.001
South Asia	179.29(132.83,234.07)	198.50(153.87,257.06)	0.36(0.16,0.56)	<0.001
Southeast Asia	44.32(32.32,56.78)	51.38(38.33,65.35)	0.48(0.42,0.54)	<0.001
Southern Latin America	117.28(95.66,142.91)	149.51(116.28,188.34)	0.77(0.41,1.12)	<0.001
Southern Sub-Saharan Africa	163.92(127.43,204.60)	149.58(119.25,186.03)	−0.30(−0.54,−0.06)	0.015
Tropical Latin America	68.12(54.74,84.07)	68.20(54.56,84.74)	0.01(−0.14,0.16)	0.869
Western Europe	209.36(170.36,255.16)	171.17(129.90,220.71)	−0.68(−0.87,−0.49)	<0.001
Western Sub-Saharan Africa	18.29(12.30,25.96)	22.31(15.17,31.50)	0.65(0.59,0.72)	<0.001

DALYs, disability-adjusted life-years. UI, uncertainty interval. AAPCs, average annual percentage changes. CI, confidence interval. ^*^The *P*-value was determined by joinpoint regression analysis.

### 3.2 Regional level

At the regional level, the greatest increases in ASIR of RA among individuals aged 60 and above from 1990 to 2021 were observed in North Africa and the Middle East (from 3.12 per 100,000 population [95% UI 1.66–4.94] in 1990 to 4.86 per 100,000 population [2.66–7.57] in 2021; AAPC 1.44 [95% CI 1.40–1.47]), Southeast Asia (from 5.94 per 100,000 population [95% UI 3.54–8.74] in 1990 to 8.64 per 100,000 population [5.27–12.52] in 2021; AAPC 1.23 [95% CI 1.18–1.28]), and Andean Latin America (from 22.33 per 100,000 population [95% UI 15.92–29.77] in 1990 to 32.47 per 100,000 population [21.75–45.07] in 2021; AAPC 1.22 [95% CI 1.17–1.27]), while High-income Asia Pacific showed the most significant decrease with an AAPC of −0.46 (95% CI −0.50 to −0.41) (Table 1). For ASPR, Andean Latin America had the largest increase (from 604.01 per 100,000 population [95% UI 539.57–674.17] in 1990 to 1058.60 per 100,000 population [949.15–1177.15] in 2021; AAPC 1.83 [95% CI 1.78–1.88]) (Table 2). Except for Central Asia and Western Sub-Saharan Africa, where the ASDR of RA among those aged 60 and above increased statistically significantly from 1990 to 2021 (AAPC 5.26 [95% CI 1.84–8.81]; AAPC 0.45 [95% CI 0.20–0.70]), all other regions experienced a decline, with the most pronounced decrease in Central Europe (from 3.18 per 100,000 population [95% UI 2.98–3.37] in 1990 to 1.47 per 100,000 population [1.31–1.471] in 2021; AAPC −2.58 [95% CI -3.21 to −1.94]) (Table 3). From 1990 to 2021, the region with the most significant increase in age-standardized DALYs rate for RA among those aged 60 and above was Central Asia (from 40.53 per 100,000 population [95% UI 28.01–56.06] in 1990 to 69.62 per 100,000 population [52.74–90.72] in 2021; AAPC 1.68 [95% CI 0.90–2.46]), while High-income Asia Pacific had the largest decrease with an AAPC of −1.24 (95% CI −1.51 to −0.97) (Table 4). Notably, in 2021, Australasia had the highest ASIR, ASPR, and age-standardized DALYs rate for RA among those aged 60 and above (407.2 per 100,000 population [95% UI 326.9–505.2]), (1478.59 per 100,000 population [95% UI 1297.40–1680.18]), and (242.95 per 100,000 population [95% UI 181.33–315.36]), respectively.

### 3.3 National level

**Figure 2:**
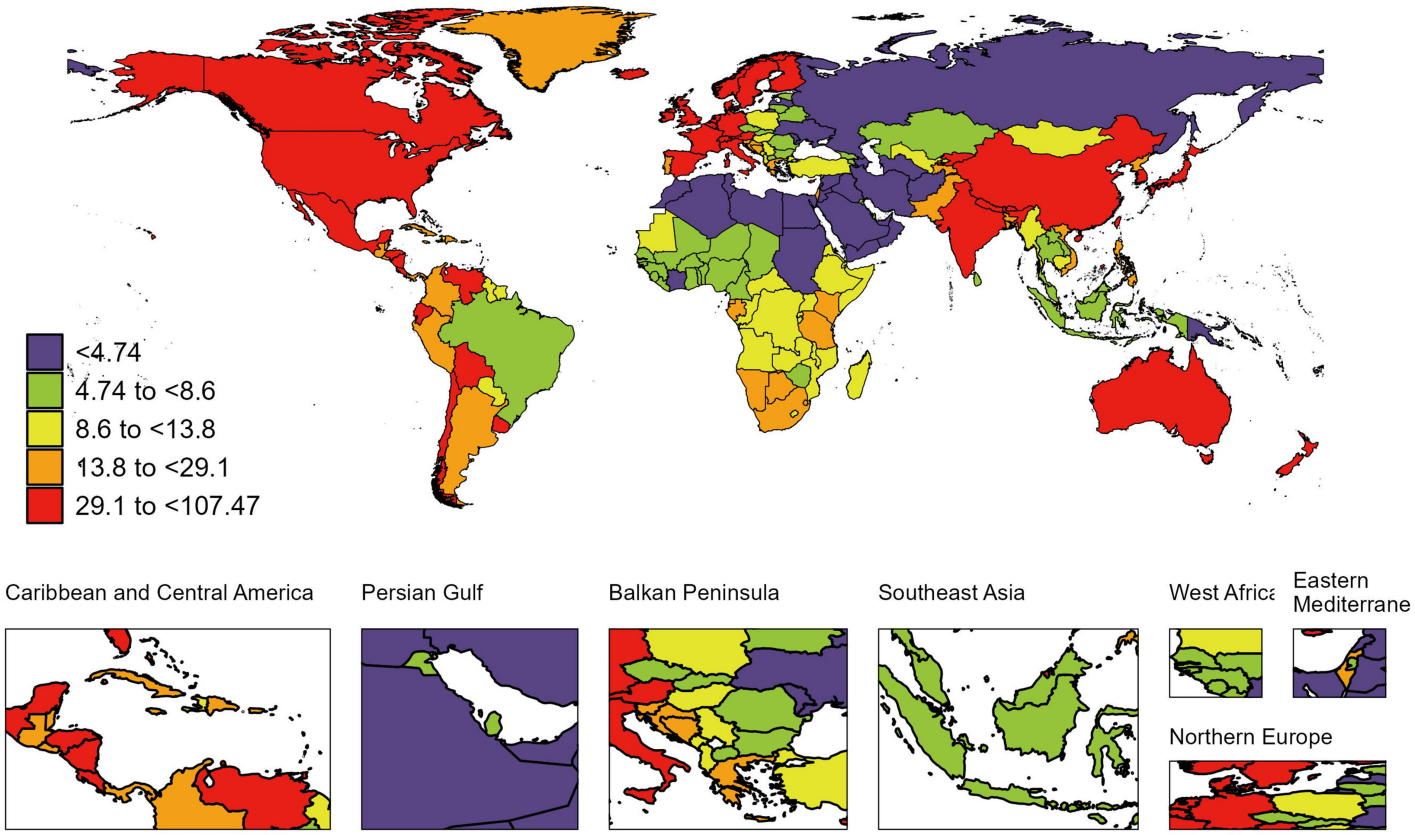
Global map of 2021 ASIR of RA among people aged 60 years and older. ASIR, age-standardized incidence rate; RA, rheumatoid arthritis. The colored boxes represent five age-standardized incidence rates intervals, categorized from lowest to highest: <4.74 (purple), 4.74 to <8.6 (chartreuse), 8.6 to <13.8 (lemon yellow), 13.8 to <29.1 (tangerine), and 29.1 to <107.47 (deep red). A color gradient from purple (lowest) to deep red (highest) corresponds to increasing incidence rates.

**Figure 3:**
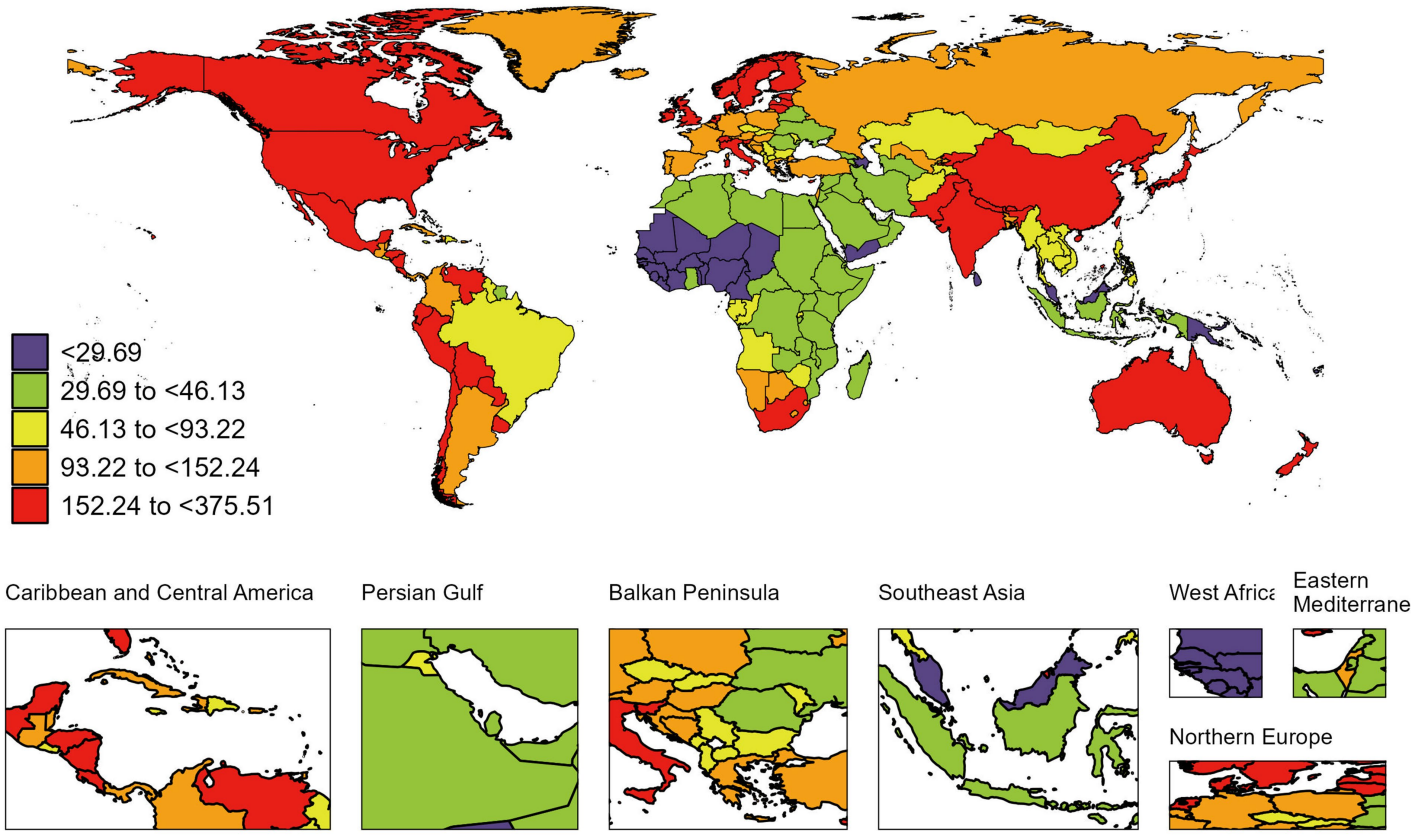
Global map of 2021 the age-standardized DALYs rate of RA among people aged 60 years and older. DALYs, disability-adjusted life-years; RA, rheumatoid arthritis. The colored boxes represent five age-standardized DALYs rates intervals, categorized from lowest to highest: <29.69(purple), 29.69 to <46.13 (chartreuse), 46.13 to <93.22 (lemon yellow), 93.22 to <152.24 (tangerine), and 152.24 to <375.51 (deep red). A color gradient from purple (lowest) to deep red (highest) corresponds to increasing DALYs rates.

At the national level, the most pronounced increase in the ASIR of RA among individuals aged 60 and above from 1990 to 2021 was observed in Viet Nam (from 7.36 per 100,000 population [95% UI 4.34–11.04] in 1990 to 14.69 per 100,000 population [8.68–21.88] in 2021; AAPC 2.25 [95% CI 2.19–2.30]). Notably, in 2021, the highest ASIR of RA among those aged 60 and above was found in Ireland (107.47 per 100,000 population [95% UI 69.87–148.72]) (Supplementary Table 1 and Figure 2). From 1990 to 2021, Equatorial Guinea experienced the greatest increase in ASPR of RA among those aged 60 and above (from 264.70 per 100,000 population [95% UI 232.02–303.43] in 1990 to 513.55 per 100,000 population [449.04–587.57] in 2021; AAPC 2.16 [95% CI 2.08–2.24]). In 2021, the country with the highest ASPR of RA among this age group was Ireland (2343.43 per 100,000 population [95% UI 2065.66–2636.13]) (Supplementary Table 2). During the same period, Turkmenistan saw the largest increase in the ASDR of RA among those aged 60 and above (from 0.01 per 100,000 population [95% UI 0.01–0.01] in 1990 to 0.56 per 100,000 population [0.40–0.82] in 2021; AAPC 14.47 [95% CI 10.02–19.10]). Notably, in 2021, the highest ASDR of RA among this age group was recorded in Honduras (11.12 per 100,000 population [95% UI 6.62–17.06]) (Supplementary Table 3). From 1990 to 2019, Mauritius experienced the greatest increase in the age-standardized DALYs rate due to RA (from 27.69 per 100,000 population [95% UI 17.10–41.01] in 1990 to 74.15 per 100,000 population [57.54–94.35] in 2021; AAPC 3.12 [95% CI 1.26–5.01]). Notably, in 2021, the highest age-standardized DALYs rate due to RA was observed in Ireland (375.51 per 100,000 population [95% UI 277.74–487.64]) (Supplementary Table 4 and Figure 3).

### 3.4 Burden of RA by age and sex

**Figure 4:**
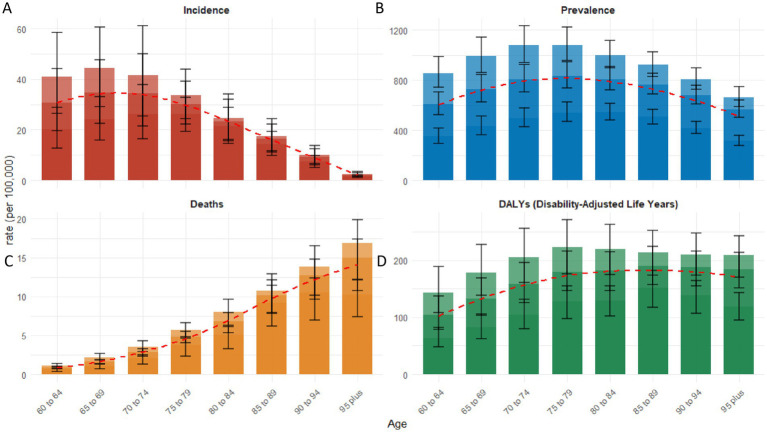
The distribution of age-specific rates of senile RA across various age groups in 2021: incidence **(A)**, prevalence **(B)**, deaths **(C)**, and DALYs **(D)**. Bar chart displaying measure values and standard deviation (error bars) for 95% uncertainty intervals. The red dashed line illustrates the trend of rate changes.

In 2021, the incidence rate of RA among individuals aged 60 and above globally increased with age, peaking in the 65–69 age group, and subsequently declined (Table 1). Similarly, the prevalence rate initially increased with age, reaching its peak in the 75–79 age group, followed by a decrease (Table 2). Notably, the deaths rate was positively correlated with population aging (Table 3). Furthermore, the DALYs rate increased with age, peaking in the 85–89 age group, after which this trend began to decline (Table 4 and Figure 4).

From 1990 to 2021, the ASIR and ASPR of RA among individuals aged ≥60 globally increased for both sexes (Figures 1A,B). The ASIR rose from 18.41 to 22.41 per 100,000 in males (AAPC 0.64, 95% CI 0.61–0.66) and from 30.39 to 37.49 in females (AAPC 0.68, 95% CI 0.65–0.71) (Table 1). Similarly, the ASPR increased from 375.29 to 445.74 in males (AAPC 0.56, 95% CI 0.54–0.58) and from 884.21 to 969.92 in females (AAPC 0.45, 95% CI 0.43–0.47) (Table 2). Females consistently exhibited higher rates than males. Conversely, the ASDR and age-standardized DALYs rate experienced a decline (Figures 1C,D). The ASDR decreased from 2.87 to 2.45 in males (AAPC −0.49, 95% CI − 0.66 to −0.31) and from 5.10 to 3.79 in females (AAPC −0.95, 95% CI − 1.12 to −0.78) (Table 3). The age-standardized DALYs rate showed smaller reductions, decreasing from 95.32 to 95.15 in males (AAPC −0.02, 95% CI − 0.12 to 0.08) and from 194.78 to 184.55 in females (AAPC −0.17, 95% CI − 0.27 to −0.07) (Table 4). Again, female rates surpassed male rates (Figures 1C,D).

### 3.5 Effects of different SDI on age-standardized DALYs rate

**Figure 5:**
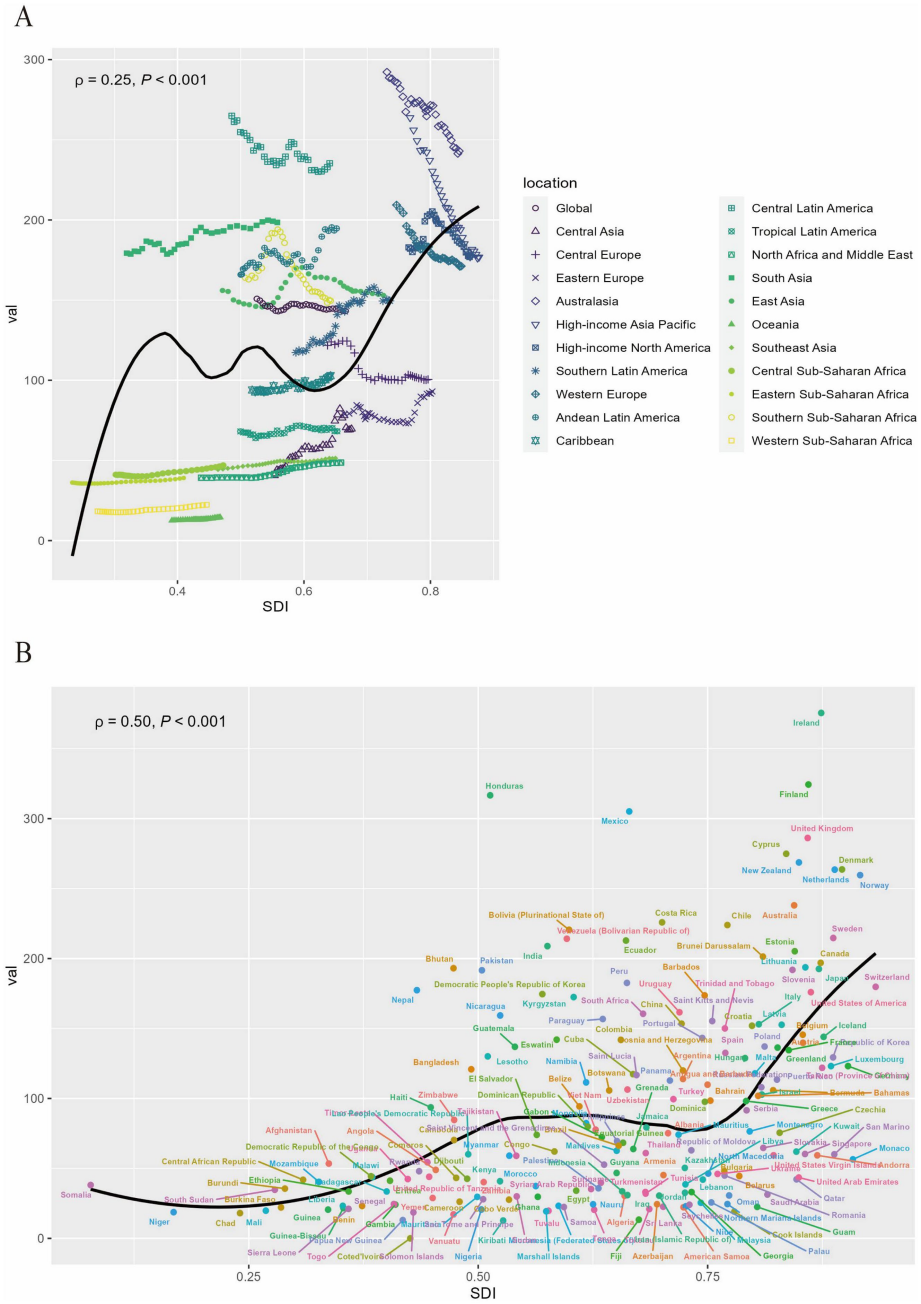
The association between the age-standardized DALYs rate of RA among people aged 60 years and older and SDI at the regional level from 1990 to 2021 **(A)** and national level at 2021 **(B)**. DALYs, disability-adjusted life-years; RA, rheumatoid arthritis; SDI, social development index.

At the regional level, the age-standardized DALYs rate exhibits a non-linear relationship with SDI. As SDI levels increase, it shows an intermittent pattern of rise and fall (Figure 5A). From 1990 to 2021, Australasia, Central Latin America, Southern Sub-Saharan Africa, and East Asia experienced a declining trend, albeit remaining generally higher than expected. Conversely, Western Sub-Saharan Africa, Central Sub-Saharan Africa, Oceania, North Africa and the Middle East, Southeast Asia, Tropical Latin America, as well as Central Asia and Eastern Europe, witnessed an upward trend, yet lower than anticipated. Analysis at the national level reveals a non-linear relationship between the age-standardized DALYs rate and SDI, with a high burden of RA not only present in developed countries but also in developing nations (Figure 5B). Notably, Honduras, Mexico, Finland, Ireland, the United Kingdom, New Zealand, Cyprus, Denmark, the Netherlands, and numerous other countries have age-standardized DALYs rates significantly higher than expected. Meanwhile, countries such as the United Arab Emirates, Qatar, Monaco, Fiji, and Kiribati have age-standardized DALYs rates lower than anticipated.

## 4 Discussion

Rheumatoid arthritis is a systemic autoimmune disease that affects multiple body systems, and if not treated promptly, it can cause severe damage to joints and their surrounding tissues. Therefore, research on the global burden of RA, particularly among the older adult population, is crucial. To our knowledge, this study is the first to comprehensively describe the incidence, prevalence, mortality, and DALYs rates of RA among individuals aged 60 and older across 204 countries. Our analysis includes global, regional, and national levels, covering data from 1990 to 2021. Compared with previous studies, this research utilizes GBD 2021 and improved methodologies, providing more accurate and comprehensive estimates of the RA burden. In particular, we have incorporated more data on individuals aged 60 and above, the primary age group for RA incidence and burden. Through cross-regional and temporal comparisons, we have uncovered the geographic diversity and locational differences in the RA burden, which can assist countries and regions in formulating more precise response strategies. Additionally, we have conducted stratified analyses of global trends by gender, age, and SDI, allowing us to focus on the disease burden across different groups for targeted interventions.

Our study reveals that in 2021, ASIR of RA for the entire population aged 60 and above was 30.32 per 100,000 people, and ASPR was 726.91 per 100,000 people. Compared with 1990, these two rates increased by 21.91 and 14.38%, respectively. Furthermore, our research highlights some significant trends in the RA burden from 1990 to 2021. Although the ASIR and ASPR of RA rose during certain periods, inflection points were also observed, particularly in recent years, where ASDR and the age-standardized DALYs rate of RA has shown a declining trend. This may be attributed to improved effectiveness of RA treatments, the promotion of early interventions, and enhanced public awareness of RA. Overall, the burden of RA remains significant globally, impacting not only the physical health and quality of life of patients but also imposing a socioeconomic burden.

An analysis of the incidence, prevalence, mortality, and DALYs rate of RA among older adult individuals across different genders and age groups has revealed that all these four indicators are higher for females compared to males, aligning with the findings of Safiri et al. (10) and Shi et al. (18). Age-stratified research indicates that in 2021, the incidence of RA among individuals aged 60 and above globally increased with age, peaking in the 65–69 age group, followed by a decline. Notably, RA mortality correlates positively with population aging. Furthermore, the prevalence and DALYs rates peak in the 75–79 and 85–89 age groups, respectively, highlighting the need for prevention and treatment strategies prior to these specific age periods. It is worth noting that this study reaffirms the substantial proportion of the global RA burden borne by females, potentially linked to sex hormone levels and their impact on the immune system. Existing research suggests that, as anti-inflammatory hormones, androgens inhibit humoral and cellular immune responses, whereas estrogens enhance humoral immune responses (19). A study on RA patients found significantly reduced concentrations of gonadal and adrenal androgens, particularly testosterone, dihydrotestosterone, and dehydroepiandrosterone sulfate, in bodily fluids (including blood, synovial fluid, smears, and saliva) (20). This finding implies that decreased levels of these immunosuppressive androgens may contribute to the adverse progression of RA. Therefore, females should pay closer attention to RA prevention and treatment, and considering therapeutic modulation to balance hormone levels could be a worthwhile direction to explore in advanced biological therapies for RA.

Upon deeper examination of the relationship between SDI and the age-standardized DALYs rate across different regions and countries, we observe a complex, non-linear association. We also find that the burden of RA is not confined to countries at specific development levels but is widespread across nations and regions with varying SDI levels. This finding corroborates with the earlier analysis by Safiri et al. (10) based on the GBD 2017, further attesting to RA as a global health concern. Additionally, variations in RA incidence and burden exist among different regions and countries, possibly attributed to environmental risk factors, discrepancies in healthcare policies, and accessibility to health services. Notably, the ASIR, ASPR, and age-standardized DALYs rate for RA among individuals aged 60 and above is highest in the Australasia region. In Australasia, which includes Australia and New Zealand, the percentage of individuals over the age of 60 significantly exceeds the global average (21). As the population ages, an increasing number of individuals develop RA, live with its effects, and experience related disabilities. Moreover, the region’s high prevalence of obesity (22)—recognized as an independent risk factor for RA—aggravates the condition. Adipose tissue in obese individuals secretes pro-inflammatory substances, such as leptin and interleukin-6, which promote immune responses, thereby intensifying autoimmune reactions and elevating the risk of RA-related complications (23). Both environmental factors and genetic predispositions play critical roles in the etiology of RA (24). Specific populations, including Indigenous Australians and New Zealand Māori, may possess susceptibility alleles, particularly in the HLA-DRB1 region (25), further predisposing them to RA. Although the high diagnostic rate in Australasia indicates a strong capacity for detection, the persistently high rates of disability suggest deficiencies in disease management, particularly concerning timely and effective interventions for high-risk groups. Conversely, while RA prevalence remains relatively low in Southeast Asia—especially in Cambodia, Myanmar, and Vietnam—the region paradoxically exhibits some of the highest annual incidence growth rates globally. This apparent contradiction likely stems from intersecting challenges: diagnostic limitations and barriers to therapeutic access within developing healthcare systems, compounded by a cultural normalization of geriatric arthralgia that delays clinical presentation. In conclusion, the complex interplay of demographic, metabolic, genetic, and healthcare access factors contributes to the regional disparities in the burden of RA. Tailored strategies—ranging from improved early diagnosis and optimized resource allocation to culturally sensitive interventions—are urgently needed to alleviate the overall health burden of RA.

A study published by the GBD 2021 Rheumatoid Arthritis Collaborators in Lancet Rheumatol predicts a continued increase in RA prevalence by 2050, underscoring the importance of prevention and treatment (5). When developing health promotion strategies, emphasis should be placed on RA risk factors such as smoking, hormones, stress, and obesity, with smoking being a well-established primary risk factor for RA that warrants precise monitoring (26, 27). A related study proposed several recommendations targeting known factors associated with RA risk to prevent its development (28). These suggestions include smoking cessation, reducing exposure to silica, dust, and occupational risks, maintaining a healthy weight, increasing leisure-time physical activity, maintaining good oral hygiene, maximizing breastfeeding where possible, striving for a high-quality diet while avoiding high-salt intake, consuming ample omega-3 fatty acids and fish, reducing consumption of sugar-sweetened soft drinks, moderate alcohol consumption, and maintaining adequate vitamin D levels. These recommendations provide valuable guidance for developing RA prevention strategies tailored to individuals aged 60 and above, especially considering the specific risks and challenges this age group may face. Future research can further explore the effectiveness of these preventive measures in reducing RA incidence and mitigating the disease burden, thereby providing a more scientific basis for public health policy formulation.

Existing literature predominantly emphasizes the significance of early diagnosis of RA, the timely initiation of treatment, and the early attainment of optimal remission targets (29, 30). Therefore, to more effectively reduce the global burden of RA, we urge governments, health agencies, and all sectors of society to increase support for early RA diagnosis and treatment. This includes, but is not limited to, enhancing public awareness of RA, strengthening screening capabilities at primary healthcare facilities, promoting effective treatment protocols, and optimizing the allocation of healthcare resources. Simultaneously, we must continually monitor advancements in RA treatment, such as the application effects of innovative therapies like biologics and small-molecule targeted drugs, to timely adjust and optimize treatment strategies. In recent years, with the development of new drugs, biologics have become an integral part of RA treatment, significantly improving disease activity and patient quality of life. Notably, JAK inhibitors tofacitinib and baricitinib are widely used in routine clinical practice (31). Although high-income regions have widely adopted biologic or targeted synthetic disease-modifying anti-rheumatic drugs (DMARDs), traditional medications like methotrexate and sulfasalazine remain effective and economical choices globally (5). It is worth mentioning that methotrexate tablets are listed in the WHO Model List of Essential Medicines (32). Although biologic or targeted synthetic DMARDs may show more pronounced efficacy (33), their high costs limit their application in low- and middle-income countries. Therefore, increasing the use of low-cost traditional DMARDs and raising awareness of early RA diagnosis and treatment are crucial for alleviating the global burden.

However, this study also has limitations. Firstly, it primarily relies on the GBD 2021 database, thus subject to variations in data quality and availability. Data from low- and middle-income countries may particularly suffer from issues of quality, comparability, accuracy, and completeness, affecting the comprehensiveness and accuracy of the findings. Secondly, variations exist in RA screening, assessment, and treatment across countries, with challenges faced particularly by less developed nations. This limits the representativeness of the statistical data and may lead to underestimation or overestimation of the RA burden in certain regions or countries. Lastly, the RA data included in GBD 2021 do not differentiate between disease stages nor contain sufficient risk factors. This constrains our in-depth analysis of the RA burden and prevents the identification of risk factors in individual regions and countries, thereby affecting the targeted nature of policy formulation.

## 5 Conclusion

This study finds that from 1990 to 2021, the global ASIR and ASPR of RA among individuals aged 60 and older showed an overall upward trend, while the ASDR and age-standardized DALYs rate declined. Despite the effectiveness of early diagnosis, preventive measures, and novel treatments in recent years, RA remains a global public health challenge. Future efforts should focus on enhancing early diagnosis, optimizing treatment, and raising public awareness. When formulating RA prevention and control policies, special attention should be given to the older adult population, especially older adult females, to mitigate the burden of RA.

## Data Availability

The original contributions presented in the study are included in the article/Supplementary material, further inquiries can be directed to the corresponding author.
