# Supplementary material for: Global, regional, and national burdens of rheumatoid arthritis among people aged 60 years and older from 1990 to 2021: a trend analysis for the Global Burden of Disease Study 2021
Source: Front Public Health. 2025 May 26;13:1527680. doi: 10.3389/fpubh.2025.1527680 (PMC12146162; doi:10.3389/fpubh.2025.1527680)
Supplement: Supplementary file 1 [file Table_1.docx]

Supplementary Material

# Supplementary Data

**Supplementary Table 1**. The ASIR and AAPCs of rheumatoid arthritis among people aged 60 years and older from 1990 to 2021.

|  | Incidence per 100000 population (95% UI)in 1990 | Incidence per 100000 population (95% UI)in 2021 | AAPCs (95% CI), 1990 to 2021 | P value * |
| --- | --- | --- | --- | --- |
| Country |  |  |  |  |
| Afghanistan | 2.05(1.09,3.23) | 2.68(1.47,4.17) | 0.87(0.85,0.89) | ＜0.001 |
| Albania | 6.40(3.51,9.88) | 11.68(6.44,17.83) | 1.97(1.88,2.06) | ＜0.001 |
| Algeria | 2.65(1.41,4.17) | 4.01(2.21,6.31) | 1.35(1.31,1.39) | ＜0.001 |
| American Samoa | 3.25(1.74,5.17) | 4.09(2.24,6.42) | 0.74(0.7,0.78) | ＜0.001 |
| Andorra | 12.71(8.04,18.3) | 13.70(8.81,19.61) | 0.25(0.21,0.29) | ＜0.001 |
| Angola | 10.02(6.31,14.39) | 12.10(7.38,17.83) | 0.61(0.59,0.64) | ＜0.001 |
| Antigua and Barbuda | 20.89(16.42,6.37) | 27.19(21.13,4.41) | 0.86(0.84,0.87) | ＜0.001 |
| Argentina | 15.51(10.28,21.54) | 16.15(10.29,23.06) | 0.13(0.11,0.15) | ＜0.001 |
| Armenia | 4.07(2.30,6.34) | 5.53(,3.11,8.54) | 1(0.96,1.04) | ＜0.001 |
| Australia | 64.07(42.98,88.12) | 74.45(50.16,102.94) | 0.48(0.44,0.51) | ＜0.001 |
| Austria | 32.75(21.24,46.42) | 32.36(20.85,44.81) | -0.04(-0.07,0) | 0.031 |
| Azerbaijan | 3.38(1.95,5.18) | 4.08（2.43，6.12） | 0.59(0.5,0.69) | ＜0.001 |
| Bahamas | 17.91(3.42,23.08) | 20.99（15.38，27.79） | 0.52(0.46,0.57) | ＜0.001 |
| Bahrain | 4.80(2.52,7.75) | 8.08（4.28，13.26） | 1.69(1.63,1.74) | ＜0.001 |
| Bangladesh | 10.30(6.85,14.13) | 15.76（10.55，21.93） | 1.38(1.3,1.46) | ＜0.001 |
| Barbados | 29.78(22.78,38.47) | 36.82（6.75，48.51） | 0.68(0.65,0.71) | ＜0.001 |
| Belarus | 3.49(1.77,5.61) | 5.33（2.76，8.68） | 1.37(1.31,1.43) | ＜0.001 |
| Belgium | 35.95(23.93,50.04) | 38.54（25.41，53.25） | 0.22(0.2,0.25) | ＜0.001 |
| Belize | 15.75(11.86,20.49) | 17.94(13.33,23.39) | 0.42(0.38,0.47) | ＜0.001 |
| Benin | 5.77(3.51,8.40) | 6.70(4.04,9.73) | 0.48(0.45,0.52) | ＜0.001 |
| Bermuda | 23.15(17.31,30.24) | 31.39(23.79,41.01) | 0.99(0.98,0.99) | ＜0.001 |
| Bhutan | 25.12(16.59,35.32 | 41.02(26.29,58.56) | 1.6(1.58,1.61) | ＜0.001 |
| Bolivia (Plurinational State of) | 25.34(17.56,35.02) | 35.82(23.00,50.61) | 1.11(1.02,1.2) | ＜0.001 |
| Bosnia and Herzegovina | 9.98(5.41,6.21) | 14.29(8.03,22.48) | 1.19(1,1.38) | ＜0.001 |
| Botswana | 12.48(7.92,17.93) | 15.16(9.36,21.88) | 0.63(0.6,0.67) | ＜0.001 |
| Brazil | 5.19(2.72,8.40) | 5.51(2.99,8.77) | 0.22(0.19,0.26) | ＜0.001 |
| Brunei Darussalam | 25.32(16.53,35.17) | 32.09(21.35,44.57) | 0.76(0.72,0.79) | ＜0.001 |
| Bulgaria | 4.39(2.41,6.85) | 6.18(3.54,9.64) | 1.12(1.08,1.17) | ＜0.001 |
| Burkina Faso | 5.75(3.45,8.39) | 6.44(3.95,9.30) | 0.37(0.33,0.42) | ＜0.001 |
| Burundi | 9.94(6.29,14.03) | 10.57(6.76,15.03) | 0.2(0.18,0.21) | ＜0.001 |
| Cabo Verde | 5.12(3.07,7.64) | 7.51(4.51,11.01) | 1.25(1.2,1.3) | ＜0.001 |
| Cambodia | 5.03(3.17,7.26) | 9.35(5.82,13.38) | 2.01(1.97,2.05) | ＜0.001 |
| Cameroon | 6.36(3.80,9.41) | 7.44(4.57,10.71) | 0.51(0.49,0.52) | ＜0.001 |
| Canada | 38.91(29.71,48.53) | 53.10(39.88,65.99) | 1.01(0.96,1.05) | ＜0.001 |
| Central African Republic | 9.27(5.74,13.47) | 9.17(5.64,13.23) | -0.03(-0.04,-0.02) | ＜0.001 |
| Chad | 5.18(3.13,7.60) | 5.51(3.39,8.01) | 0.2(0.17,0.23) | ＜0.001 |
| Chile | 28.57(17.95,42.07) | 47.18(28.88,70.15) | 1.63(1.58,1.68) | ＜0.001 |
| China | 22.79(13.23,4.75) | 29.59(18.26,43.32) | 0.85(0.83,0.88) | ＜0.001 |
| Colombia | 13.19(8.41,19.03) | 16.87(10.60,24.46) | 0.8(0.75,0.86) | ＜0.001 |
| Comoros | 11.79(7.63,16.73) | 14.17(8.76,20.27) | 0.58(0.54,0.63) | ＜0.001 |
| Congo | 11.23(6.77,16.42) | 13.32(8.07,19.67) | 0.55(0.49,0.61) | ＜0.001 |
| Cook Islands | 2.65(1.39,4.23) | 3.45(1.87,5.56) | 0.86(0.81,0.91) | ＜0.001 |
| Costa Rica | 21.82(13.78,31.91) | 29.64(18.13,43.77) | 0.98(0.89,1.08) | ＜0.001 |
| Croatia | 10.92(5.81,7.68) | 18.49(9.81,28.99) | 1.73(1.66,1.81) | ＜0.001 |
| Cuba | 13.58(8.76,19.46) | 16.93(10.65,24.56) | 0.71(0.65,0.77) | ＜0.001 |
| Cyprus | 63.12(40.37,89.53) | 80.39(50.44,113.62) | 0.76(0.65,0.86) | ＜0.001 |
| Czechia | 5.31(2.87,8.42) | 7.95(4.37,12.45) | 1.31(1.28,1.33) | ＜0.001 |
| Democratic People's Republic of Korea | 17.31(10.68,25.17) | 23.74(15.12,34.47) | 1.02(0.98,1.07) | ＜0.001 |
| Democratic Republic of the Congo | 10.19(6.47,14.56) | 11.16(6.93,16.27) | 0.29(0.24,0.34) | ＜0.001 |
| Denmark | 63.79(45.04,84.65) | 87.28(60.51,17.23) | 1.01(0.99,1.04) | ＜0.001 |
| Djibouti | 11.39(7.13,16.39) | 13.23(8.12,19.13) | 0.49(0.47,0.51) | ＜0.001 |
| Dominica | 20.56(16.32,5.60) | 23.51(8.84,29.35) | 0.43(0.41,0.46) | ＜0.001 |
| Dominican Republic | 19.97(16.48,23.85) | 23.24(19.00,28.26) | 0.5(0.47,0.54) | ＜0.001 |
| Ecuador | 35.97(26.99,46.10) | 49.52(34.56,66.67) | 1.01(0.93,1.09) | ＜0.001 |
| Egypt | 2.54(1.34,4.03) | 3.86(2.08,6.06) | 1.36(1.34,1.39) | ＜0.001 |
| El Salvador | 9.73(6.95,13.01) | 13.81(9.53,18.86) | 1.15(1.08,1.22) | ＜0.001 |
| Equatorial Guinea | 10.21(6.39,14.55) | 16.53(9.72,25.16) | 1.57(1.47,1.66) | ＜0.001 |
| Eritrea | 10.21(6.59,14.4) | 12.39(7.69,17.81) | 0.63(0.61,0.65) | ＜0.001 |
| Estonia | 5.47(2.66,9.23) | 7.28(3.55,12.62) | 0.93(0.85,1) | ＜0.001 |
| Eswatini | 13.65(8.56,19.93) | 15.12(9.34,22.19) | 0.34(0.31,0.37) | ＜0.001 |
| Ethiopia | 10.63(6.25,15.91) | 11.12(6.79,16.15) | 0.14(0.12,0.17) | ＜0.001 |
| Fiji | 2.19(1.16,3.52) | 2.41(1.28,3.8) | 0.32(0.26,0.38) | ＜0.001 |
| Finland | 63.67(45.94,84.03) | 78.38(54.81,104.1) | 0.67(0.65,0.7) | ＜0.001 |
| France | 35.28(23.42,48.58) | 42.73(28.15,58.65) | 0.62(0.58,0.65) | ＜0.001 |
| Gabon | 12.96(7.83,19.15) | 16.13(9.62,4.12) | 0.71(0.69,0.72) | ＜0.001 |
| Gambia | 5.84(3.51,8.59) | 7.21(4.37,10.48) | 0.68(0.65,0.7) | ＜0.001 |
| Georgia | 4.34(2.48,6.72) | 5.00(2.87,7.64) | 0.45(0.42,0.48) | ＜0.001 |
| Germany | 25.89(16.96,36.65) | 29.43(19.91,41.5) | 0.41(0.38,0.44) | ＜0.001 |
| Ghana | 5.28(3.01,8.04) | 7.03(4.11,10.47) | 0.92(0.85,1) | ＜0.001 |
| Greece | 16.09(10.35,23.06) | 18.88(12.41,26.56) | 0.51(0.49,0.54) | ＜0.001 |
| Greenland | 15.74(10.25,22.22) | 27.21(7.43,38.34) | 1.78(1.69,1.87) | ＜0.001 |
| Grenada | 8.89(6.23,12.19) | 13.22(9.27,17.93) | 1.29(1.25,1.34) | ＜0.001 |
| Guam | 3.11(1.67,4.99) | 3.99(2.19,6.29) | 0.83(0.72,0.93) | ＜0.001 |
| Guatemala | 15.49(11.93,19.69) | 24.57(18.33,32.22) | 1.5(1.47,1.52) | ＜0.001 |
| Guinea | 5.54(3.35,8.05) | 6.15(3.75,8.88) | 0.34(0.3,0.38) | ＜0.001 |
| Guinea-Bissau | 5.55(3.38,7.97) | 6.52(4.05,9.31) | 0.52(0.5,0.53) | ＜0.001 |
| Guyana | 7.29(5.19,9.75) | 10.09(7.49,13.26) | 1.06(1.03,1.09) | ＜0.001 |
| Haiti | 11.51(8.81,14.75) | 12.81(9.67,16.78) | 0.35(0.33,0.36) | ＜0.001 |
| Honduras | 25.92(17.98,35.36) | 33.25(22.12,47.32) | 0.8(0.74,0.87) | ＜0.001 |
| Hungary | 9.34(4.84,15.01) | 12.24(6.48,18.75) | 0.89(0.8,0.98) | ＜0.001 |
| Iceland | 30.35(19.58,42.92) | 36.05(23.17,50.5) | 0.55(0.5,0.6) | ＜0.001 |
| India | 39.49(25.18,56.43) | 56.21(35.92,79.43) | 1.15(1.09,1.2) | ＜0.001 |
| Indonesia | 3.78(2.13,5.72) | 5.56(3.35,8.15) | 1.25(1.17,1.32) | ＜0.001 |
| Iran (Islamic Republic of) | 2.78(1.42,4.53) | 3.83(2.04,6.12) | 1.03(0.99,1.07) | ＜0.001 |
| Iraq | 2.92(1.54,4.67) | 4.49(2.47,7.06) | 1.39(1.37,1.42) | ＜0.001 |
| Ireland | 77.08(49.64,105.48) | 107.47(69.87,148.72) | 1.09(1.05,1.14) | ＜0.001 |
| Israel | 20.69(14.07,28.61) | 28.83(19.58,39.32) | 1.08(1.06,1.1) | ＜0.001 |
| Italy | 38.02(22.54,56.83) | 42.37(26.28,61.37) | 0.35(0.34,0.36) | ＜0.001 |
| Jamaica | 13.36(9.84,17.6) | 15.79(11.70,20.85) | 0.54(0.53,0.55) | ＜0.001 |
| Japan | 49(29.63,73.13) | 42.04(25.98,61.41) | -0.49(-0.51,-0.47) | ＜0.001 |
| Jordan | 2.89(1.54,4.60) | 4.29(2.33,6.73) | 1.27(1.21,1.33) | ＜0.001 |
| Kazakhstan | 3.96(2.28,6.12) | 5.27(3.07,8.08) | 0.93(0.86,1) | ＜0.001 |
| Kenya | 13.09（7.91，19.33） | 14.50(8.89,21.17) | 0.32(0.31,0.34) | ＜0.001 |
| Kiribati | 2.01（1.08，3.16） | 2.28(1.24,3.52) | 0.41(0.37,0.44) | ＜0.001 |
| Kuwait | 3.61（1.87，5.86） | 5.12(2.74,8.17) | 1.14(1.09,1.19) | ＜0.001 |
| Kyrgyzstan | 19.83（12.35，28.81） | 32.58(20.04,48.48) | 1.62(1.58,1.65) | ＜0.001 |
| Lao People's Democratic Republic | 4.94（3.10，6.98） | 8.40(5.29,12.08) | 1.74(1.72,1.76) | ＜0.001 |
| Latvia | 3.51（1.73，5.88） | 4.66(2.36,7.73) | 0.91(0.81,1.02) | ＜0.001 |
| Lebanon | 2.94（1.55，4.70） | 4.22(2.26,6.72) | 1.18(1.15,1.21) | ＜0.001 |
| Lesotho | 11.92（7.58，17.00） | 13.35(8.56,19.16) | 0.37(0.34,0.4) | ＜0.001 |
| Liberia | 5.26(3.09,7.78) | 6.53(3.94,9.59) | 0.7(0.64,0.75) | ＜0.001 |
| Libya | 2.60(1.36,4.13) | 3.43(1.85,5.49) | 0.9(0.8,1) | ＜0.001 |
| Lithuania | 4.51(2.18,7.58) | 6.10(3.05,10.21) | 0.98(0.94,1.01) | ＜0.001 |
| Luxembourg | 27.11(18.05,38.25) | 32.4221.4245.35 | 0.57(0.53,0.61) | ＜0.001 |
| Madagascar | 9.31(6.06,13.09) | 10.67(6.92,14.96) | 0.44(0.43,0.45) | ＜0.001 |
| Malawi | 11.44(7.36,16.29) | 13.51(8.54,19.62) | 0.53(0.5,0.56) | ＜0.001 |
| Malaysia | 3.72(2.17,5.65) | 4.88(2.89,7.43) | 0.88(0.82,0.94) | ＜0.001 |
| Maldives | 5.06(3.01,7.55) | 9.34(5.58,14.06) | 2.01(1.96,2.06) | ＜0.001 |
| Mali | 4.96(2.96,7.33) | 5.62(3.46,8.13) | 0.4(0.37,0.43) | ＜0.001 |
| Malta | 23.28(15.23,32.63) | 27.53(18.26,38.4) | 0.55(0.51,0.59) | ＜0.001 |
| Marshall Islands | 3.06(1.69,4.73) | 3.74(2.09,5.68) | 0.65(0.62,0.69) | ＜0.001 |
| Mauritania | 6.15(3.69,8.90) | 8.79(5.32,12.69) | 1.16(1.1,1.21) | ＜0.001 |
| Mauritius | 7.39(4.23,11.28) | 10.92(6.41,16.54) | 1.26(1.22,1.3) | ＜0.001 |
| Mexico | 50.63(30.86,74.39) | 45.28(27.98,66.6) | -0.38(-0.43,-0.32) | ＜0.001 |
| Micronesia (Federated States of) | 3.08(1.69,4.82) | 4.01(2.20,6.18) | 0.87(0.82,0.92) | ＜0.001 |
| Monaco | 12.46(7.82,18.42) | 13.36(8.62,19.24) | 0.23(0.14,0.33) | ＜0.001 |
| Mongolia | 7.35(4.98,10.26) | 10.01(6.41,14.29) | 1(0.97,1.03) | ＜0.001 |
| Montenegro | 8.26(4.26,13.28) | 10.58(5.76,16.56) | 0.81(0.78,0.83) | ＜0.001 |
| Morocco | 2.33(1.22,3.70) | 3.34(1.84,5.22) | 1.18(1.15,1.2) | ＜0.001 |
| Mozambique | 11.22(7.31,5.72) | 12.91(8.18,18.38) | 0.44(0.4,0.48) | ＜0.001 |
| Myanmar | 5.11(3.19,7.46) | 9.01(5.59,13.09) | 1.84(1.79,1.88) | ＜0.001 |
| Namibia | 12.28(7.85,17.42) | 15.49(9.63,22.6) | 0.75(0.73,0.78) | ＜0.001 |
| Nauru | 3.34(1.83,5.20) | 4.40(2.42,6.79) | 0.9(0.85,0.94) | ＜0.001 |
| Nepal | 21.13(14.16,29.40) | 34.22(22.09,47.55) | 1.56(1.52,1.6) | ＜0.001 |
| Netherlands | 79.68(52.34,110.84) | 82.72(55.01,114.08) | 0.13(0.08,0.19) | ＜0.001 |
| New Zealand | 76.57(46.11,115.22) | 74.99(46.44,109.17) | -0.06(-0.08,-0.04) | ＜0.001 |
| Nicaragua | 28.68(22.05,36.51) | 38.99(29.32,51.03) | 1(0.95,1.06) | ＜0.001 |
| Niger | 4.83(2.92,7.09) | 5.49(3.37,7.89) | 0.42(0.36,0.48) | ＜0.001 |
| Nigeria | 4.50(2.57,6.84) | 5.21(3.06,7.81) | 0.48(0.45,0.5) | ＜0.001 |
| Niue | 3.33(1.79,5.29) | 4.27(2.35,6.70) | 0.81(0.8,0.82) | ＜0.001 |
| North Macedonia | 4.57(2.47,7.19) | 6.85(3.77,10.71) | 1.32(1.29,1.35) | ＜0.001 |
| Northern Mariana Islands | 3.91(2.09,6.18) | 4.53(2.49,7.01) | 0.48(0.35,0.6) | ＜0.001 |
| Norway | 83.72(51.34,123.63) | 75.81(47.11,110.27) | -0.32(-0.34,-0.3) | ＜0.001 |
| Oman | 2.48(1.31,4.00) | 3.92(2.13,6.08) | 1.48(1.46,1.51) | ＜0.001 |
| Pakistan | 17.47(10.65,25.75) | 17.42(10.86,25.16) | -0.01(-0.03,0.01) | 0.474 |
| Palau | 3.72(2.00，5.81) | 4.71(2.55,7.53) | 0.78(0.75,0.81) | ＜0.001 |
| Palestine | 3.21(1.72,5.07) | 4.91(2.72,7.67) | 1.37(1.33,1.41) | ＜0.001 |
| Panama | 13.40(8.89,18.44) | 16.89(11.15,23.88) | 0.74(0.69,0.8) | ＜0.001 |
| Papua New Guinea | 2.36(1.27,3.72) | 2.68(1.51,4.09) | 0.43(0.38,0.48) | ＜0.001 |
| Paraguay | 6.44(3.45,10.36) | 9.01(4.78,14.75) | 1.08(1.03,1.13) | ＜0.001 |
| Peru | 15.30(9.97,21.64) | 22.79(13.62,34.21) | 1.29(1.23,1.35) | ＜0.001 |
| Philippines | 13.45(8.28,19.48) | 14.02(8.98,19.81) | 0.16(0.12,0.2) | ＜0.001 |
| Poland | 8.97(4.52,14.94) | 10.68(5.77,17.03) | 0.57(0.54,0.6) | ＜0.001 |
| Portugal | 19.59(12.78,27.24) | 28.97(18.97,40.53) | 1.26(1.22,1.31) | ＜0.001 |
| Puerto Rico | 17.61(1.92,4.65) | 23.04(14.74,33.59) | 0.88(0.82,0.94) | ＜0.001 |
| Qatar | 3.67(1.91,5.82) | 6.38(3.39,10.31) | 1.8(1.75,1.84) | ＜0.001 |
| Republic of Korea | 19.04(12.81,26.05) | 31.63(21.48,42.28) | 1.63(1.56,1.7) | ＜0.001 |
| Republic of Moldova | 3.04(1.55,4.92) | 4.30(2.25,6.99) | 1.13(1.1,1.17) | ＜0.001 |
| Romania | 4.45(2.41,7.10) | 7.26(4.07,11.34) | 1.59(1.56,1.62) | ＜0.001 |
| Russian Federation | 2.77(1.31,4.68) | 3.38(1.68,5.59) | 0.64(0.61,0.67) | ＜0.001 |
| Rwanda | 10.70(6.94,15.34) | 13.72(8.51,9.76) | 0.8(0.77,0.83) | ＜0.001 |
| Saint Kitts and Nevis | 19.69(14.79,25.46) | 25.59(18.54,34.51) | 0.85(0.81,0.9) | ＜0.001 |
| Saint Lucia | 22.82(18.58,28.02) | 28.39(22.86,34.98) | 0.71(0.68,0.74) | ＜0.001 |
| Saint Vincent and the Grenadines | 10.97(8.11,4.59) | 12.60(9.38,16.58) | 0.44(0.42,0.47) | ＜0.001 |
| Samoa | 3.25(1.76,5.15) | 4.14(2.28,6.43) | 0.8(0.74,0.85) | ＜0.001 |
| San Marino | 12.49(7.79,18.49) | 12.71(8.02,18.77) | 0.07(-0.01,0.15) | 0.098 |
| Sao Tome and Principe | 5.98(3.57,8.73) | 8.04(4.92,11.68) | 0.96(0.94,0.99) | ＜0.001 |
| Saudi Arabia | 2.47(1.30,3.98) | 3.77(2.06,5.88) | 1.37(1.33,1.41) | ＜0.001 |
| Senegal | 6.03（3.61，8.78） | 7.52(4.61,10.85) | 0.71(0.66,0.75) | ＜0.001 |
| Serbia | 8.49(4.89,13.14） | 11.29(6.25,17.75) | 0.92(0.88,0.97) | ＜0.001 |
| Seychelles | 3.59(2.08,5.45) | 4.75(2.80,7.19) | 0.91(0.88,0.93) | ＜0.001 |
| Sierra Leone | 5.55(3.33,8.16) | 6.26(3.81,8.98) | 0.39(0.32,0.47) | ＜0.001 |
| Singapore | 10.30(6.54,14.93) | 13.75(8.74,19.67) | 0.94(0.88,1) | ＜0.001 |
| Slovakia | 4.79(2.60,7.58) | 6.97(3.94,10.93) | 1.21(1.19,1.24) | ＜0.001 |
| Slovenia | 17.78(9.05,27.42) | 25.35(14.22,38.46) | 1.13(1.03,1.23) | ＜0.001 |
| Solomon Islands | 2.49(1.33,3.88) | 3.03（1.69，4.65） | 0.64(0.61,0.67) | ＜0.001 |
| Somalia | 10.10(6.51,14.43) | 10.59（6.83，14.98） | 0.15(0.14,0.17) | ＜0.001 |
| South Africa | 19.14(11.24,29.26) | 19.51（1.62，9.47） | 0.07(0.03,0.11) | 0.001 |
| South Sudan | 9.77(6.26,13.92) | 11.10（7.06，15.79） | 0.41(0.38,0.44) | ＜0.001 |
| Spain | 26.48(19.23,34.00) | 34.95（28.12，42.99） | 0.9(0.88,0.93) | ＜0.001 |
| Sri Lanka | 3.51(2.06,5.31) | 4.88（2.88，7.26） | 1.07(1.04,1.1) | ＜0.001 |
| Sudan | 2.09(1.10,3.34) | 2.92（1.60，4.52） | 1.08(1.03,1.12) | ＜0.001 |
| Suriname | 8.79(6.48,11.52) | 10.08（7.34，13.22） | 0.44(0.42,0.47) | ＜0.001 |
| Sweden | 73.86(49.23,104.68) | 67.39（45.40，92.89） | -0.3(-0.32,-0.28) | ＜0.001 |
| Switzerland | 58.38(38.39,82.04) | 62.39（40.79，86.69） | 0.21(0.18,0.24) | ＜0.001 |
| Syrian Arab Republic | 2.78(1.46,4.41) | 4.18（2.28，6.55） | 1.33(1.27,1.39) | ＜0.001 |
| Taiwan (Province of China) | 21.91(13.88,30.92) | 26.82（20.48，33.40） | 0.67(0.61,0.73) | ＜0.001 |
| Tajikistan | 9.90(6.38,14.29) | 14.36（9.69，20.21） | 1.19(1.14,1.25) | ＜0.001 |
| Thailand | 5.72(3.29,8.63) | 7.97（4.55，12.31） | 1.08(1.05,1.1) | ＜0.001 |
| Timor-Leste | 5.12(3.13,7.46) | 8.56（5.48，12.26） | 1.67(1.57,1.76) | ＜0.001 |
| Togo | 6.05(3.55,8.76) | 7.19（4.39，10.27） | 0.57(0.51,0.64) | ＜0.001 |
| Tokelau | 2.84(1.55,4.45) | 3.87（2.15，5.97） | 1.02(0.94,1.09) | ＜0.001 |
| Tonga | 3.09(1.69,4.93) | 3.77（2.06，5.93） | 0.65(0.62,0.67) | ＜0.001 |
| Trinidad and Tobago | 24.63(19.16,31.42) | 29.49（22.78，37.71） | 0.58(0.55,0.6) | ＜0.001 |
| Tunisia | 2.62(1.38,4.20) | 3.80（2.09，6.02） | 1.21(1.16,1.26) | ＜0.001 |
| Turkey | 5.25(2.81,8.29) | 8.76（4.69，13.77） | 1.67(1.63,1.7) | ＜0.001 |
| Turkmenistan | 3.33(1.89,5.13) | 4.04（2.33，6.17） | 0.63(0.61,0.65) | ＜0.001 |
| Tuvalu | 2.84(1.58,4.40) | 3.77（2.09，5.82） | 0.92(0.87,0.96) | ＜0.001 |
| Uganda | 10.86(6.84,15.35) | 13.19(8.22,18.93) | 0.63(0.6,0.66) | ＜0.001 |
| Ukraine | 3.16(1.58,5.21) | 3.92(2.04,6.32) | 0.69(0.66,0.73) | ＜0.001 |
| United Arab Emirates | 3.19(1.68,4.99) | 4.11(2.22,6.53) | 0.82(0.79,0.86) | ＜0.001 |
| United Kingdom | 67.99(44.03,96.15) | 75.61(49.87,105.79) | 0.34(0.32,0.36) | ＜0.001 |
| United Republic of Tanzania | 11.80(7.51,17.09) | 13.99(8.71,20.19) | 0.55(0.53,0.56) | ＜0.001 |
| United States of America | 40.24(25.98,56.54) | 47.82(31.49,65.95) | 0.57(0.53,0.6) | ＜0.001 |
| United States Virgin Islands | 15.07(11.11,9.72) | 18.01(13.00,23.78) | 0.57(0.55,0.6) | ＜0.001 |
| Uruguay | 26.41(19.22,34.78) | 30.86(21.48,41.97) | 0.51(0.47,0.54) | ＜0.001 |
| Uzbekistan | 7.5(4.20,11.78) | 8.83(4.97,14.00) | 0.53(0.49,0.58) | ＜0.001 |
| Vanuatu | 2.82(1.55,4.36) | 3.38(1.88,5.24) | 0.58(0.53,0.62) | ＜0.001 |
| Venezuela (Bolivarian Republic of) | 23.86(16.48,33.12) | 29.31(9.41,41.71) | 0.65(0.6,0.7) | ＜0.001 |
| Viet Nam | 7.36(4.34,11.04) | 14.69(8.68,21.88) | 2.25(2.19,2.3) | ＜0.001 |
| Yemen | 2.06(1.08,3.30) | 2.64(1.45,4.07) | 0.8(0.77,0.83) | ＜0.001 |
| Zambia | 7.83(4.99,11.1) | 9.52(5.98,13.78) | 0.63(0.6,0.67) | ＜0.001 |
| Zimbabwe | 7.56(5.13,10.41) | 7.43(5.04,10.29) | -0.06(-0.08,-0.03) | ＜0.001 |
| UI = uncertainty interval. AAPCs = average annual percentage changes. CI = confidence interval. * The P-value was determined by joinpoint regression analysis. | | | | |
|  |  |  |  |  |

**Supplementary Table 2**. The ASPR and AAPCs of rheumatoid arthritis among people aged 60 years and older from 1990 to 2021.

|  | Incidence per 100000 population (95% UI)in 1990 | Incidence per 100000 population (95% UI)in 2021 | AAPCs (95% CI), 1990 to 2021 | P value * |
| --- | --- | --- | --- | --- |
| Country |  |  |  |  |
| Afghanistan | 91.92(79.04,107.6) | 140.96(123.66,160.7) | 1.39(1.35，1.44) | ＜0.001 |
| Albania | 249.57(209.15,296.26) | 438.68(373.2,513.94) | 1.84(1.74，1.94) | ＜0.001 |
| Algeria | 110.33(92.83,131.47) | 174.05(147.74,204.94) | 1.48(1.46，1.51) | ＜0.001 |
| American Samoa | 140.32(118.48,166.13) | 175.41(150.7,204.14) | 0.72(0.71，0.74) | ＜0.001 |
| Andorra | 387.48(327.74,453.81) | 429.13(364.99,503.15) | 0.33(0.3，0.37) | ＜0.001 |
| Angola | 261.16(228.56,297.54) | 351.55(308.67,398.86) | 0.96(0.93，0.99) | ＜0.001 |
| Antigua and Barbuda | 343.25(299.27,393.01) | 482.02(423.12,548.15) | 1.1(1.06，1.15) | ＜0.001 |
| Argentina | 407.94(354.24,468.42) | 581.83(517.86,652.57) | 1.15(1.13，1.17) | ＜0.001 |
| Armenia | 174.64(146.99,207.63) | 267.68(231.46,309.46) | 1.39(1.36，1.42) | ＜0.001 |
| Australia | 1307.3(1148.15,1476.64) | 1447.85(1267.6,1644.79) | 0.33(0.3，0.36) | ＜0.001 |
| Austria | 911.58(807.57,1032.61) | 880.79(773.78,1001.17) | -0.11(-0.15，-0.06) | ＜0.001 |
| Azerbaijan | 144.12(120.03,173.67) | 181.15(153.19,214.52) | 0.74(0.61，0.86) | ＜0.001 |
| Bahamas | 348.32(304.46,395.41) | 441.46(389.02,501.78) | 0.77(0.67，0.87) | ＜0.001 |
| Bahrain | 303.12(266.5,345.21) | 569.72(508.98,635.89) | 2.05(1.98，2.12) | ＜0.001 |
| Bangladesh | 222.38(188.88,259.84) | 366.46(315.74,426.62) | 1.63(1.6，1.66) | ＜0.001 |
| Barbados | 542.84(476.29,614.96) | 745.79(664.88,842.1) | 1.03(1.01，1.05) | ＜0.001 |
| Belarus | 204.83(171.94,241.19) | 334.29(287.71,384.57) | 1.58(1.54，1.63) | ＜0.001 |
| Belgium | 827.41(721.96,944.22) | 896.65(785.14,1029.96) | 0.26(0.24，0.28) | ＜0.001 |
| Belize | 314.46(275.59,358.07) | 409.7(362.72,462.91) | 0.85(0.83，0.87) | ＜0.001 |
| Benin | 139.9(117.35,166.09) | 178.01(151.79,208.05) | 0.78(0.76，0.8) | ＜0.001 |
| Bermuda | 418.26(361.4,485.08) | 584.31(506.18,675.03) | 1.09(1.04，1.13) | ＜0.001 |
| Bhutan | 443.83(379.58,521.66) | 745.79(640.55,869.79) | 1.7(1.66，1.74) | ＜0.001 |
| Bolivia (Plurinational State of) | 549.78(484.65,623.12) | 941.99(837.35,1063.72) | 1.76(1.72，1.8) | ＜0.001 |
| Bosnia and Herzegovina | 488.94(424.6,561.92) | 747.22(657.89,855.53) | 1.4(1.23，1.58) | ＜0.001 |
| Botswana | 356(313.64,403.49) | 485.72(431.1,546.12) | 1(0.93，1.07) | ＜0.001 |
| Brazil | 306.2(263.22,354.93) | 307.07(266.9,353.98) | -0.01(-0.05，0.04) | 0.722 |
| Brunei Darussalam | 611.45(536.26,698.74) | 810.05(717.91,915.03) | 0.91(0.89，0.94) | ＜0.001 |
| Bulgaria | 229.92(196.31,270.65) | 312.77(269.17,363.62) | 1(0.95，1.04) | ＜0.001 |
| Burkina Faso | 135.97(113.87,162) | 164.63(138.99,193.78) | 0.62(0.59，0.65) | ＜0.001 |
| Burundi | 231.49(201.03,265.01) | 256.5(223.52,295.74) | 0.33(0.31，0.35) | ＜0.001 |
| Cabo Verde | 142.55(119.93,168.53) | 215.12(182.62,252.27) | 1.34(1.3，1.38) | ＜0.001 |
| Cambodia | 131.53(112.5,152.91) | 237.32(203.49,274.2) | 1.92(1.9，1.94) | ＜0.001 |
| Cameroon | 162.44(137.03,190.96) | 200.2(171.02,233.71) | 0.67(0.63，0.72) | ＜0.001 |
| Canada | 905.83(855.93,955.93) | 1202.41(1138.19,1271.93) | 0.92(0.89，0.94) | ＜0.001 |
| Central African Republic | 278.18(246.16,314.21) | 285.41(253.06,322.26) | 0.07(0.04，0.11) | ＜0.001 |
| Chad | 121.64(101.49,143.97) | 133.64(113.04,156.63) | 0.3(0.28，0.32) | ＜0.001 |
| Chile | 712.26(626.15,809.24) | 1304.46(1153.44,1473.73) | 1.97(1.96，1.99) | ＜0.001 |
| China | 646.92(547.58,765.67) | 748.39(639.9,876.48) | 0.47(0.46，0.48) | ＜0.001 |
| Colombia | 435.89(386.38,492.35) | 672.72(602.88,750.49) | 1.41(1.37，1.46) | ＜0.001 |
| Comoros | 256.48(220.47,298.05) | 320.37(277.19,370.41) | 0.72(0.68，0.75) | ＜0.001 |
| Congo | 350.18(307.53,397.92) | 454.49(403.94,512.26) | 0.84(0.82，0.86) | ＜0.001 |
| Cook Islands | 105.9(85.54,130.26) | 149.51(124.06,181.27) | 1.12(1.07，1.17) | ＜0.001 |
| Costa Rica | 689.95(615.25,777.03) | 1154.71(1037.24,1291.38) | 1.68(1.64，1.71) | ＜0.001 |
| Croatia | 560.13(493.25,636.06) | 924.69(818.38,1046.16) | 1.63(1.6，1.66) | ＜0.001 |
| Cuba | 386.1(338.07,442.88) | 558.04(492.45,632.08) | 1.2(1.17，1.23) | ＜0.001 |
| Cyprus | 1233.24(1066.61,1426.32) | 1572.11(1360.62,1800.96) | 0.78(0.74，0.83) | ＜0.001 |
| Czechia | 320.04(276.55,369.15) | 463.12(404.83,530.47) | 1.19(1.13，1.25) | ＜0.001 |
| Democratic People's Republic of Korea | 527.21(463.16,600.65) | 718.38(639.4,810.7) | 1(0.96，1.04) | ＜0.001 |
| Democratic Republic of the Congo | 256.86(223.89,293.38) | 311.89(272.52,358) | 0.62(0.58，0.65) | ＜0.001 |
| Denmark | 1185.93(1049.7,1335.93) | 1593.72(1408.45,1803.37) | 0.96(0.94，0.99) | ＜0.001 |
| Djibouti | 246.57(211.04,289.53) | 314.15(270.66,362.29) | 0.79(0.77，0.81) | ＜0.001 |
| Dominica | 331.18(290.02,379.96) | 391.9(344.35,445.03) | 0.55(0.52，0.58) | ＜0.001 |
| Dominican Republic | 244.89(211.15,283.6) | 317.51(273.8,369.39) | 0.85(0.82，0.87) | ＜0.001 |
| Ecuador | 647.55(572.58,728.86) | 1093.25(975.15,1226.54) | 1.71(1.67，1.76) | ＜0.001 |
| Egypt | 125.39(106.8,147.23) | 206.95(179.5,238.94) | 1.62(1.59，1.66) | ＜0.001 |
| El Salvador | 241.17(208.56,278.48) | 395.22(342.78,455.27) | 1.61(1.54，1.69) | ＜0.001 |
| Equatorial Guinea | 264.7(232.02,303.43) | 513.55(449.04,587.57) | 2.16(2.08，2.24) | ＜0.001 |
| Eritrea | 239.71(209.02,275.65) | 299.4(262.05,342.61) | 0.72(0.68，0.76) | ＜0.001 |
| Estonia | 767.42(706.27,833.83) | 1003.27(927.82,1087.82) | 0.84(0.76，0.92) | ＜0.001 |
| Eswatini | 431.6(379.57,488.34) | 502.64(447.63,564.2) | 0.49(0.45，0.52) | ＜0.001 |
| Ethiopia | 263.68(221.8,313.97) | 247.84(210.8,292.84) | -0.2(-0.22，-0.17) | ＜0.001 |
| Fiji | 84.59(68.67,104.68) | 101.39(84.31,122.78) | 0.6(0.53，0.66) | ＜0.001 |
| Finland | 1503.71(1342.32,1688.72) | 1880.77(1671.78,2108.97) | 0.73(0.67，0.79) | ＜0.001 |
| France | 702.92(608.48,810.81) | 835.66(722.23,967.79) | 0.56(0.51，0.6) | ＜0.001 |
| Gabon | 376.65(329.06,429.47) | 540.15(478.27,608.4) | 1.17(1.15，1.18) | ＜0.001 |
| Gambia | 142.93(118.93,170.9) | 184.52(156.51,216.8) | 0.83(0.8，0.86) | ＜0.001 |
| Georgia | 185.85(155.58,223.44) | 221.94(189.31,258.98) | 0.58(0.54，0.61) | ＜0.001 |
| Germany | 657.98(570.68,755.84) | 739.45(640.55,846.19) | 0.37(0.35，0.4) | ＜0.001 |
| Ghana | 131.56(107.07,159.39) | 187.67(155.4,224.11) | 1.16(1.13，1.18) | ＜0.001 |
| Greece | 523.98(450.99,608.78) | 610.76(530.84,701.24) | 0.49(0.48，0.51) | ＜0.001 |
| Greenland | 421.1(370.3,479.65) | 722.91(641.8,817.28) | 1.77(1.72，1.81) | ＜0.001 |
| Grenada | 190.93(161.72,224.46) | 304.39(263.12,351.44) | 1.51(1.48，1.55) | ＜0.001 |
| Guam | 127.1(105.62,153.26) | 172.26(146.43,202.22) | 1(0.86，1.14) | ＜0.001 |
| Guatemala | 302.75(266.15,343.36) | 574.34(513.92,643.54) | 2.09(2.03，2.16) | ＜0.001 |
| Guinea | 131.74(110.16,156.23) | 154.23(131.46,181.59) | 0.52(0.49，0.54) | ＜0.001 |
| Guinea-Bissau | 129.79(108.74,153.58) | 162.21(138.84,188.79) | 0.72(0.7，0.75) | ＜0.001 |
| Guyana | 156.38(132.95,184.83) | 219.2(189.89,253.89) | 1.1(1.08，1.12) | ＜0.001 |
| Haiti | 208.24(184.11,237.03) | 261.22(231.15,294.28) | 0.73(0.7，0.76) | ＜0.001 |
| Honduras | 659.5(587.07,737.51) | 1071.34(964.08,1193.09) | 1.58(1.55，1.61) | ＜0.001 |
| Hungary | 560.35(506.02,620.83) | 715.65(645.2,794.09) | 0.79(0.76，0.82) | ＜0.001 |
| Iceland | 769.05(666.41,883.85) | 892.58(766.24,1029.1) | 0.48(0.43，0.52) | ＜0.001 |
| India | 599.5(494.59,728.52) | 924.54(774.19,1106) | 1.41(1.38，1.44) | ＜0.001 |
| Indonesia | 98.4(80.58,119.59) | 129.46(108.05,154.57) | 0.89(0.86，0.91) | ＜0.001 |
| Iran (Islamic Republic of) | 135.95(115.17,160.57) | 191.03(164.38,223.28) | 1.1(1.07，1.13) | ＜0.001 |
| Iraq | 144.75(122.17,171.22) | 220.17(190.12,255.07) | 1.37(1.33，1.4) | ＜0.001 |
| Ireland | 1905.03(1693.04,2142.37) | 2343.43(2065.66,2636.13) | 0.68(0.63，0.72) | ＜0.001 |
| Israel | 484.2(411.42,564.03) | 654.43(561.09,760.45) | 0.97(0.96，0.99) | ＜0.001 |
| Italy | 930.63(778.36,1112.47) | 903.62(761.3,1077.6) | -0.09(-0.11，-0.08) | ＜0.001 |
| Jamaica | 261.16(226.32,303.14) | 355.17(311.11,405.59) | 1(0.96，1.04) | ＜0.001 |
| Japan | 1315.05(1104.91,1568.46) | 1069.09(910.11,1258.62) | -0.66(-0.74，-0.58) | ＜0.001 |
| Jordan | 139.42(119.47,163.92) | 221.32(190.34,258.16) | 1.51(1.45，1.57) | ＜0.001 |
| Kazakhstan | 171.49(144.11,204.07) | 238.28(203.06,278.16) | 1.07(1.01，1.13) | ＜0.001 |
| Kenya | 273.89(229.06,328.6) | 304.28(258.05,360.14) | 0.34(0.32，0.36) | ＜0.001 |
| Kiribati | 79.84(66.41,95.28) | 94.56(80.44,111.62) | 0.56(0.49，0.62) | ＜0.001 |
| Kuwait | 246.41(210.68,288.35) | 361.06(315.24,410.42) | 1.29(1.05，1.53) | ＜0.001 |
| Kyrgyzstan | 647.53(571.07,731.06) | 997.14(875.63,1129.77) | 1.4(1.36，1.44) | ＜0.001 |
| Lao People's Democratic Republic | 128.44(110.59,148.61) | 214.74(184.82,247.92) | 1.68(1.65，1.7) | ＜0.001 |
| Latvia | 555.63(514.23,602.73) | 692.01(638.68,749.5) | 0.7(0.63，0.77) | ＜0.001 |
| Lebanon | 189.11(162.19,222.2) | 292.35(251.47,337.62) | 1.42(1.39，1.45) | ＜0.001 |
| Lesotho | 359.17(315.59,405.61) | 426.32(379.54,478.7) | 0.55(0.49，0.6) | ＜0.001 |
| Liberia | 135.35(112.68,161.15) | 180.1(153.28,210.29) | 0.93(0.87，0.99) | ＜0.001 |
| Libya | 130.31(110.41,155.5) | 186.64(160.08,216.78) | 1.17(1.14，1.2) | ＜0.001 |
| Lithuania | 655.56(605.09,710.93) | 817.69(756.75,885.08) | 0.72(0.67，0.76) | ＜0.001 |
| Luxembourg | 673.17(585.3,775.84) | 780.57(678.06,897.41) | 0.48(0.46，0.5) | ＜0.001 |
| Madagascar | 201.43(172.92,234.27) | 247.39(216.31,285.29) | 0.67(0.65，0.69) | ＜0.001 |
| Malawi | 251.48(217.07,292.57) | 319.38(277.68,367.26) | 0.77(0.73，0.8) | ＜0.001 |
| Malaysia | 103.93(84.68,126.29) | 143.39(120.13,171.42) | 1.05(1，1.1) | ＜0.001 |
| Maldives | 183.54(159.68,210.3) | 336.02(293.37,384.23) | 1.98(1.91，2.04) | ＜0.001 |
| Mali | 121.05(100.72,144.43) | 146.76(124.44,171.89) | 0.62(0.59，0.65) | ＜0.001 |
| Malta | 599.72(519.58,685.72) | 710.45(616.04,816.65) | 0.55(0.53，0.57) | ＜0.001 |
| Marshall Islands | 116.84(100.9,135.51) | 150.15(130.45,171.68) | 0.81(0.78，0.85) | ＜0.001 |
| Mauritania | 155.03(130.94,182.7) | 227.73(194.05,265.32) | 1.25(1.22，1.28) | ＜0.001 |
| Mauritius | 198.16(165.23,238.05) | 304.34(258.82,359.42) | 1.4(1.37，1.43) | ＜0.001 |
| Mexico | 1267.5(1074.8,1486.22) | 1335.24(1159.74,1542.16) | 0.17(0.16，0.18) | ＜0.001 |
| Micronesia (Federated States of) | 123.23(105.89,143.35) | 180.16(157.81,205.61) | 1.23(1.21，1.24) | ＜0.001 |
| Monaco | 390.72(327.98,467.73) | 420.58(354.11,495.12) | 0.24(0.16，0.32) | ＜0.001 |
| Mongolia | 220.93(191.23,253.85) | 355.89(310.4,406.33) | 1.56(1.46，1.66) | ＜0.001 |
| Montenegro | 357.35(302.73,418.43) | 450.8(385.32,524.91) | 0.76(0.71，0.81) | ＜0.001 |
| Morocco | 110.42(93.7,130.47) | 171.93(148.46,199.42) | 1.44(1.42，1.46) | ＜0.001 |
| Mozambique | 235.58(203.05,274.18) | 293.06(254,336.57) | 0.71(0.69，0.73) | ＜0.001 |
| Myanmar | 134.78(114.74,157.7) | 234.15(201.76,271.23) | 1.8(1.78，1.82) | ＜0.001 |
| Namibia | 337.03(296.56,384.39) | 455.3(401.96,517.12) | 0.97(0.94，1.01) | ＜0.001 |
| Nauru | 133.68(114.42,154.91) | 191.97(167.04,219.27) | 1.17(1.14，1.2) | ＜0.001 |
| Nepal | 366.32(313.48,428.59) | 632.22(545.1,734.95) | 1.78(1.76，1.8) | ＜0.001 |
| Netherlands | 1606(1409.2,1825.93) | 1561.64(1355.7,1791.41) | -0.09(-0.14，-0.05) | ＜0.001 |
| New Zealand | 1791.5(1488.71,2142.18) | 1638.62(1382.66,1952.14) | -0.28(-0.32，-0.25) | ＜0.001 |
| Nicaragua | 517.55(455.96,586.22) | 790.66(700.21,899.17) | 1.38(1.36，1.4) | ＜0.001 |
| Niger | 116.23(96.58,138.52) | 138.96(117.66,163.79) | 0.59(0.53，0.64) | ＜0.001 |
| Nigeria | 131.9(108.7,158.96) | 156.41(131.39,185.79) | 0.56(0.51，0.61) | ＜0.001 |
| Niue | 131.83(112.05,155.59) | 180.85(155.23,209.45) | 1.03(1，1.05) | ＜0.001 |
| North Macedonia | 218.64(183.16,261.79) | 326.17(275.54,383.24) | 1.3(1.28，1.33) | ＜0.001 |
| Northern Mariana Islands | 162.52(138.63,191.69) | 189.39(162.45,220) | 0.51(0.39，0.62) | ＜0.001 |
| Norway | 2006.72(1670.81,2411.94) | 1628.06(1372.22,1946.06) | -0.67(-0.72，-0.61) | ＜0.001 |
| Oman | 111.92(92.91,134.52) | 199.8(168.6,235.32) | 1.9(1.87，1.92) | ＜0.001 |
| Pakistan | 428.02(364.89,502.04) | 404.88(345.64,474.54) | -0.14(-0.17，-0.1) | ＜0.001 |
| Palau | 151.39(129.72,176.74) | 202.24(173.56,232.94) | 0.94(0.89，0.99) | ＜0.001 |
| Palestine | 153.15(131.05,179.03) | 228.01(196.04,264.98) | 1.3(1.25，1.34) | ＜0.001 |
| Panama | 387.94(341.48,440.06) | 539.23(475.99,609.16) | 1.07(1.05，1.08) | ＜0.001 |
| Papua New Guinea | 84.44(70.64,101.31) | 98.12(83.28,115.58) | 0.48(0.45，0.51) | ＜0.001 |
| Paraguay | 394.12(345.34,448.65) | 697.24(628.99,775.27) | 1.85(1.8，1.9) | ＜0.001 |
| Peru | 600.07(532.79,673.51) | 1077.4(962.43,1205.61) | 1.91(1.8，2.02) | ＜0.001 |
| Philippines | 288.24(241.1,343.27) | 270.96(228.3,320.67) | -0.22(-0.24，-0.19) | ＜0.001 |
| Poland | 798.14(697.84,914.91) | 721.33(632.6,820.6) | -0.32(-0.35，-0.29) | ＜0.001 |
| Portugal | 567.32(503.4,641.42) | 836.02(738.36,942.29) | 1.26(1.22，1.29) | ＜0.001 |
| Puerto Rico | 395.36(342.84,457.3) | 631.24(549.34,724.05) | 1.53(1.51，1.54) | ＜0.001 |
| Qatar | 162.4(135.83,195.18) | 307.52(259.02,361.57) | 2.11(1.96，2.25) | ＜0.001 |
| Republic of Korea | 524.79(463.43,592.58) | 819.05(722.68,923.24) | 1.43(1.3，1.55) | ＜0.001 |
| Republic of Moldova | 202.02(173.35,236.27) | 301.48(262.67,345.03) | 1.29(1.24，1.35) | ＜0.001 |
| Romania | 215.4(178.6,257.57) | 349.16(298.01,409.77) | 1.57(1.54，1.59) | ＜0.001 |
| Russian Federation | 381.82(337.12,431.58) | 429.64(383.6,479.57) | 0.38(0.36，0.41) | ＜0.001 |
| Rwanda | 252.05(220.55,289.75) | 354.96(311.07,406.23) | 1.11(1.05，1.17) | ＜0.001 |
| Saint Kitts and Nevis | 368.84(324.93,418.97) | 555.69(490.69,630.08) | 1.33(1.31，1.36) | ＜0.001 |
| Saint Lucia | 351.15(309.1,398.6) | 470.15(414.91,529.9) | 0.95(0.91，0.98) | ＜0.001 |
| Saint Vincent and the Grenadines | 211.98(180.74,248.17) | 257.99(221.64,299.89) | 0.64(0.62，0.66) | ＜0.001 |
| Samoa | 132.53(113.41,155.92) | 173.3(150.2,199.69) | 0.87(0.84，0.9) | ＜0.001 |
| San Marino | 382.66(316.79,456.79) | 399.28(333.38,477.2) | 0.14(0.06，0.23) | ＜0.001 |
| Sao Tome and Principe | 156.25(131.33,185.43) | 215.65(183.13,251.44) | 1.05(1.03，1.07) | ＜0.001 |
| Saudi Arabia | 110.49(92.26,132.14) | 186.56(157.7,220.97) | 1.71(1.67，1.75) | ＜0.001 |
| Senegal | 144.84(121.28,171.35) | 187.78(158.8,220.84) | 0.84(0.83，0.86) | ＜0.001 |
| Serbia | 347.45(310.67,388.37) | 527.03(459.22,600.84) | 1.35(1.33，1.37) | ＜0.001 |
| Seychelles | 118.97(98.8,143.34) | 162.1(136.94,191.23) | 1(0.99，1.01) | ＜0.001 |
| Sierra Leone | 133.65(110.98,158.79) | 159.83(135.23,187.39) | 0.58(0.55，0.61) | ＜0.001 |
| Singapore | 310.74(264.74,363.66) | 418.93(355.2,489.02) | 0.96(0.92，1) | ＜0.001 |
| Slovakia | 251.23(215.76,292.02) | 368.29(321.87,422.07) | 1.25(1.2，1.29) | ＜0.001 |
| Slovenia | 829.17(736.89,931.86) | 1183.88(1053.85,1319.62) | 1.15(1.12，1.19) | ＜0.001 |
| Solomon Islands | 102.88(88.62,119.35) | 141.46(123.41,161.5) | 1.03(1.01，1.05) | ＜0.001 |
| Somalia | 250.65(218.76,286.54) | 260.65(228.66,297.11) | 0.13(0.12，0.14) | ＜0.001 |
| South Africa | 728.96(635.86,837.43) | 675.71(591.1,776.64) | -0.25(-0.27，-0.22) | ＜0.001 |
| South Sudan | 218.64(188.28,254.69) | 256.51(222.65,295.88) | 0.52(0.48，0.56) | ＜0.001 |
| Spain | 663.55(600.49,733.48) | 833.94(769.23,909.33) | 0.74(0.72，0.75) | ＜0.001 |
| Sri Lanka | 103.1(84.32,126.13) | 148.11(123,177.69) | 1.18(1.14，1.22) | ＜0.001 |
| Sudan | 91.03(76.89,107.92) | 139.51(119.67,161.76) | 1.39(1.37，1.41) | ＜0.001 |
| Suriname | 169.83(143.56,199.57) | 216.59(185.69,254.05) | 0.79(0.77，0.81) | ＜0.001 |
| Sweden | 1493.8(1261.34,1771.78) | 1312.18(1115.94,1547.5) | -0.42(-0.43，-0.41) | ＜0.001 |
| Switzerland | 1108.4(957.26,1292.81) | 1179.1(1017.49,1366.68) | 0.19(0.16，0.23) | ＜0.001 |
| Syrian Arab Republic | 125.83(106.07,150.44) | 199.67(170.54,235.25) | 1.51(1.47，1.55) | ＜0.001 |
| Taiwan (Province of China) | 576.94(505,655) | 645.14(618.8,674.94) | 0.37(0.29，0.46) | ＜0.001 |
| Tajikistan | 288.5(251.32,328.94) | 382.21(335.95,434.57) | 0.9(0.85，0.94) | ＜0.001 |
| Thailand | 195.86(167.73,228.44) | 292.88(252.23,340.12) | 1.31(1.27，1.34) | ＜0.001 |
| Timor-Leste | 128.26(108.96,150.81) | 205.9(176.59,239.49) | 1.54(1.51，1.57) | ＜0.001 |
| Togo | 143.89(120.23,171.38) | 184.73(157.77,215.98) | 0.81(0.76，0.86) | ＜0.001 |
| Tokelau | 109.39(92.97,128.96) | 163.39(140.57,189.64) | 1.3(1.24，1.37) | ＜0.001 |
| Tonga | 122.72(103.64,145.98) | 158.64(136.14,185.88) | 0.84(0.81，0.86) | ＜0.001 |
| Trinidad and Tobago | 472.57(419.69,531.15) | 630.91(560.97,705.48) | 0.94(0.92，0.96) | ＜0.001 |
| Tunisia | 123.72(104.55,146.88) | 193.65(165.52,225.57) | 1.46(1.42，1.5) | ＜0.001 |
| Turkey | 314.04(279.7,354.95) | 546.32(488.19,612.38) | 1.8(1.75，1.85) | ＜0.001 |
| Turkmenistan | 142.66(120.19,169.68) | 190.76(164.85,221.72) | 0.95(0.93，0.97) | ＜0.001 |
| Tuvalu | 107.01(91.52,125.42) | 153.35(132.66,177.78) | 1.17(1.15，1.19) | ＜0.001 |
| Uganda | 241.82(208.81,281.25) | 312.83(271.84,358.58) | 0.83(0.81，0.86) | ＜0.001 |
| Ukraine | 177.25(148.06,213.03) | 225.26(191.2,265.27) | 0.78(0.75，0.8) | ＜0.001 |
| United Arab Emirates | 167.1(143.31,194.61) | 200.16(172.87,232.27) | 0.58(0.49，0.67) | ＜0.001 |
| United Kingdom | 1528.28(1316.68,1790.07) | 1653.85(1435,1917.59) | 0.25(0.22，0.28) | ＜0.001 |
| United Republic of Tanzania | 258.65(222.2,301.52) | 327.85(284.18,378.02) | 0.77(0.72，0.82) | ＜0.001 |
| United States of America | 939.77(821.97,1078.14) | 1084.19(951.79,1233.93) | 0.47(0.44，0.5) | ＜0.001 |
| United States Virgin Islands | 279.41(238.18,326.69) | 361.32(311.41,421.2) | 0.83(0.8，0.86) | ＜0.001 |
| Uruguay | 499.55(436.26,572.44) | 692.03(606.46,784.5) | 1.06(0.99，1.13) | ＜0.001 |
| Uzbekistan | 518.18(467.69,572.05) | 624.28(567.99,688.2) | 0.61(0.56，0.66) | ＜0.001 |
| Vanuatu | 103.24(87.61,121.52) | 131.64(113.26,151.73) | 0.78(0.76，0.8) | ＜0.001 |
| Venezuela (Bolivarian Republic of) | 667.69(601.94,745.05) | 958.93(867.81,1061.38) | 1.15(1.07，1.23) | ＜0.001 |
| Viet Nam | 211.52(180.01,248.94) | 403.58(347.36,467.27) | 2.11(2.09，2.13) | ＜0.001 |
| Yemen | 90.95(76.96,108.22) | 120.42(103.03,140.58) | 0.91(0.88，0.95) | ＜0.001 |
| Zambia | 219.82(193.12,250.46) | 294.72(260.45,334.22) | 0.96(0.94，0.98) | ＜0.001 |
| Zimbabwe | 231.34(201.95,263.86) | 232.62(205.87,263.3) | 0.01(-0.05，0.08) | 0.664 |
| UI = uncertainty interval. AAPCs = average annual percentage changes. CI = confidence interval. * The P-value was determined by joinpoint regression analysis. | | | | |
|  |  |  |  |  |

**Supplementary Table 3.** The Deaths rate and AAPCs of rheumatoid arthritis among people aged 60 years and older from 1990 to 2021.

|  | Incidence per 100000 population (95% UI)in 1990 | Incidence per 100000 population (95% UI)in 2021 | AAPCs (95% CI), 1990 to 2021 | P value * |
| --- | --- | --- | --- | --- |
| country |  |  |  |  |
| Afghanistan | 1.06(0.43,2.19) | 1.79(0.79,3.48) | 1.7(1.58,1.83) | ＜0.001 |
| Albania | 2.41(1.55,3.5) | 1.18(0.67,1.89) | -2.34(-2.94,-1.73) | ＜0.001 |
| Algeria | 0.31(0.14,0.58) | 0.46(0.23,0.82) | 1.33(1.01,1.65) | ＜0.001 |
| American Samoa | 0.02(0.01,0.04) | 0.01(0,0.01) | -4.38(-8.75,0.2) | 0.061 |
| Andorra | 0.6(0.32,0.98) | 0.28(0.15,0.47) | -2.44(-2.83,-2.05) | ＜0.001 |
| Angola | 0.42(0.1,3.85) | 0.27(0.06,2.88) | -1.37(-1.48,-1.27) | ＜0.001 |
| Antigua and Barbuda | 2.95(2.35,3.66) | 3.74(2.97,4.61) | 0.96(-0.33,2.27) | ＜0.001 |
| Argentina | 2.28(1.89,2.73) | 2.21(1.78,2.7) | -0.14(-0.92,0.64) | 0.144 |
| Armenia | 0.04(0.03,0.05) | 0.78(0.64,0.92) | 9.31(3.35,15.62) | 0.722 |
| Australia | 6.77(5.64,7.93) | 3.55(2.83,4.33) | -2.03(-2.96,-1.09) | 0.002 |
| Austria | 4.19(3.42,5.05) | 1.7(1.33,2.1) | -2.96(-4.05,-1.85) | ＜0.001 |
| Azerbaijan | 0.02(0.01,0.04) | 0.08(0.02,0.12) | 4.14(3.37,4.92) | ＜0.001 |
| Bahamas | 3.38(2.6,4.29) | 3.32(2.51,4.3) | -0.01(-1.48,1.5) | 0.994 |
| Bahrain | 0.24(0.13,0.44) | 1.7(0.94,2.82) | 6.22(4.84,7.61) | ＜0.001 |
| Bangladesh | 6.61(3.5,11.46) | 4.76(2.49,8.36) | -0.95(-1.53,-0.36) | 0.001 |
| Barbados | 5.61(4.48,6.98) | 6.07(4.53,7.89) | 0.42(-0.91,1.76) | 0.54 |
| Belarus | 0.09(0.08,0.11) | 0.11(0.08,0.15) | 0.56(-0.24,1.37) | 0.169 |
| Belgium | 3.52(2.86,4.22) | 1.96(1.52,2.42) | -1.63(-2.26,-0.99) | ＜0.001 |
| Belize | 2.22(1.73,2.89) | 2.94(2.26,3.68) | 0.94(0.17,1.72) | 0.017 |
| Benin | 0.06(0.02,0.13) | 0.04(0.01,0.08) | -1.66(-1.88,-1.45) | ＜0.001 |
| Bermuda | 3.43(2.65,4.32) | 2.61(1.88,3.49) | -0.63(-1.23,-0.03) | 0.04 |
| Bhutan | 7.65(3.67,12.9) | 6.23(3.02,10.77) | -0.64(-0.69,-0.58) | ＜0.001 |
| Bolivia (Plurinational State of) | 8.8(5.5,13.3) | 7.13(4.22,11.11) | -0.67(-0.8,-0.55) | ＜0.001 |
| Bosnia and Herzegovina | 2.29(1.4,3.4) | 1.46(0.89,2.25) | -1.35(-1.57,-1.14) | ＜0.001 |
| Botswana | 3.86(1.76,6.36) | 2.73(0.75,4.63) | -1.18(-2.09,-0.27) | 0.012 |
| Brazil | 1.64(1.47,1.8) | 1.5(1.3,1.67) | -0.36(-0.98,0.26) | 0.256 |
| Brunei Darussalam | 8.62(5.24,13.19) | 5.75(3.37,9.06) | -1.4(-1.64,-1.15) | ＜0.001 |
| Bulgaria | 0.86(0.73,1.01) | 0.46(0.36,0.58) | -1.96(-3.02,-0.88) | ＜0.001 |
| Burkina Faso | 0.09(0.03,0.21) | 0.05(0.02,0.11) | -1.82(-2.24,-1.4) | ＜0.001 |
| Burundi | 0.3(0.08,2.53) | 0.17(0.04,1.61) | -1.83(-1.89,-1.76) | ＜0.001 |
| Cabo Verde | 0.05(0.01,0.1) | 0.01(0,0.03) | -4.09(-4.45,-3.72) | ＜0.001 |
| Cambodia | 2.56(1.28,4) | 2.69(1.28,4.2) | 0.18(0.12,0.25) | ＜0.001 |
| Cameroon | 0.08(0.03,0.16) | 0.04(0.02,0.09) | -2.17(-2.42,-1.92) | ＜0.001 |
| Canada | 4.52(3.73,5.36) | 2.75(2.17,3.36) | -1.62(-2.43,-0.8) | ＜0.001 |
| Central African Republic | 0.55(0.13,5.17) | 0.36(0.07,3.7) | -1.34(-1.48,-1.2) | ＜0.001 |
| Chad | 0.07(0.02,0.15) | 0.05(0.02,0.12) | -0.88(-1.05,-0.71) | ＜0.001 |
| Chile | 6.21(5.13,7.34) | 3.82(3.06,4.66) | -1.6(-3.12,-0.05) | 0.043 |
| China | 4.51(3.56,5.72) | 3.81(2.61,4.76) | -0.52(-1,-0.03) | 0.039 |
| Colombia | 5.32(4.51,6.19) | 3.31(2.53,4.2) | -1.54(-2.67,-0.39) | 0.009 |
| Comoros | 0.26(0.07,2.1) | 0.17(0.04,1.67) | -1.28(-1.44,-1.12) | ＜0.001 |
| Congo | 0.55(0.15,5.26) | 0.29(0.07,3.06) | -2.04(-2.19,-1.89) | ＜0.001 |
| Cook Islands | 0(0,0) | 0(0,0) | -2.04(-2.88,-1.19) | ＜0.001 |
| Costa Rica | 5.29(4.35,6.29) | 5.08(3.93,6.23) | -0.27(-0.42,-0.12) | 0.001 |
| Croatia | 2.96(2.52,3.44) | 2.06(1.65,2.51) | -1.17(-1.91,-0.43) | 0.002 |
| Cuba | 2.94(2.38,3.5) | 3.05(2.39,3.8) | 0.14(-0.27,0.55) | 0.502 |
| Cyprus | 15.03(8.44,24.95) | 5.15(3.04,8.08) | -3.41(-4.14,-2.67) | ＜0.001 |
| Czechia | 1.54(1.3,1.8) | 0.97(0.78,1.2) | -1.42(-2.32,-0.51) | 0.002 |
| Democratic People's Republic of Korea | 4.91(2.97,7.73) | 4.98(3.06,7.77) | 0.05(0,0.11) | 0.048 |
| Democratic Republic of the Congo | 0.41(0.1,3.78) | 0.3(0.06,3.21) | -1.04(-1.17,-0.9) | ＜0.001 |
| Denmark | 4.41(3.61,5.3) | 4.03(3.19,4.93) | -0.21(-1.03,0.61) | 0.612 |
| Djibouti | 0.23(0.06,1.9) | 0.16(0.04,1.49) | -1.08(-1.17,-1) | ＜0.001 |
| Dominica | 4.1(2.57,6.11) | 3.7(2.26,5.63) | -0.32(-0.41,-0.24) | ＜0.001 |
| Dominican Republic | 5.92(3.08,8.87) | 3.43(1.94,5.41) | -1.73(-2.26,-1.19) | ＜0.001 |
| Ecuador | 8.16(7,9.46) | 5.49(4.28,6.91) | -1.44(-4.06,1.24) | 0.289 |
| Egypt | 0.65(0.34,1.06) | 0.41(0.25,0.63) | -1.5(-1.92,-1.08) | ＜0.001 |
| El Salvador | 1.78(1.06,2.83) | 1.5(0.91,2.35) | -0.42(-0.8,-0.04) | 0.032 |
| Equatorial Guinea | 0.51(0.12,4.99) | 0.24(0.06,2.48) | -2.38(-2.5,-2.26) | ＜0.001 |
| Eritrea | 0.29(0.07,2.58) | 0.21(0.05,2.26) | -0.95(-1,-0.91) | ＜0.001 |
| Estonia | 4.12(3.36,4.93) | 3.92(3.14,4.81) | -0.13(-1.22,0.98) | 0.818 |
| Eswatini | 5.51(1.84,9.04) | 4.58(0.98,8.51) | -0.58(-0.78,-0.37) | ＜0.001 |
| Ethiopia | 0.34(0.1,2.59) | 0.14(0.04,1.29) | -2.76(-2.87,-2.64) | ＜0.001 |
| Fiji | 0(0,0.01) | 0(0,0) | -3.04(-3.37,-2.71) | ＜0.001 |
| Finland | 13.44(11.16,15.88) | 5.37(4.29,6.45) | -2.84(-3.26,-2.41) | ＜0.001 |
| France | 3.94(3.22,4.76) | 1.84(1.4,2.28) | -2.46(-2.95,-1.97) | ＜0.001 |
| Gabon | 0.5(0.13,4.6) | 0.29(0.07,2.94) | -1.76(-2.04,-1.47) | ＜0.001 |
| Gambia | 0.06(0.02,0.14) | 0.04(0.01,0.09) | -1.2(-1.5,-0.9) | ＜0.001 |
| Georgia | 0.02(0.02,0.03) | 0.31(0.26,0.37) | 8.23(-3.3,21.13) | 0.169 |
| Germany | 3.85(3.08,4.68) | 1.84(1.41,2.28) | -2.31(-3.35,-1.26) | ＜0.001 |
| Ghana | 0.04(0.01,0.1) | 0.46(0.14,1) | 7.92(7.12,8.73) | ＜0.001 |
| Greece | 1.19(0.99,1.41) | 1.21(0.96,1.47) | -0.09(-0.99,0.81) | 0.843 |
| Greenland | 3.09(1.86,4.94) | 2.31(1.26,4.32) | -0.85(-1.18,-0.53) | ＜0.001 |
| Grenada | 1.49(1.07,1.99) | 2.04(1.51,2.66) | 1.08(0.4,1.77) | 0.002 |
| Guam | 0.01(0,0.01) | 0(0,0) | -6.48(-10.39,-2.41) | 0.002 |
| Guatemala | 4.36(3.73,5.09) | 4.17(3.38,5.05) | -0.39(-1.09,0.3) | 0.268 |
| Guinea | 0.06(0.02,0.14) | 0.04(0.02,0.1) | -1.21(-1.3,-1.13) | ＜0.001 |
| Guinea-Bissau | 0.09(0.03,0.19) | 0.06(0.02,0.12) | -1.54(-1.69,-1.38) | ＜0.001 |
| Guyana | 0.15(0.11,0.2) | 1.32(0.95,1.76) | 7.22(4.8,9.69) | ＜0.001 |
| Haiti | 5.32(2.55,9.91) | 4(1.83,7.9) | -0.91(-1,-0.82) | ＜0.001 |
| Honduras | 8.88(4.57,16.53) | 11.12(6.62,17.06) | 0.74(0.43,1.05) | ＜0.001 |
| Hungary | 4.01(3.35,4.74) | 2.05(1.57,2.55) | -2.07(-2.64,-1.48) | ＜0.001 |
| Iceland | 3.51(2.82,4.24) | 1.84(1.41,2.33) | -2.3(-3,-1.59) | ＜0.001 |
| India | 6.49(4.24,9.65) | 5.82(4.08,8.82) | -0.31(-1,0.39) | 0.384 |
| Indonesia | 0.79(0.32,1.18) | 1.1(0.41,1.62) | 1.1(0.97,1.22) | ＜0.001 |
| Iran (Islamic Republic of) | 0.31(0.19,0.47) | 0.34(0.23,0.46) | 0.31(0.08,0.55) | 0.009 |
| Iraq | 0.24(0.11,0.44) | 0.17(0.08,0.3) | -1.13(-1.29,-0.97) | ＜0.001 |
| Ireland | 10.7(9.11,12.54) | 4.93(3.9,5.97) | -2.32(-2.94,-1.71) | ＜0.001 |
| Israel | 1.78(1.43,2.18) | 1.23(0.95,1.53) | -1.13(-1.8,-0.45) | 0.001 |
| Italy | 2.9(2.57,3.15) | 2.48(2.04,2.79) | -0.53(-1.52,0.47) | 0.299 |
| Jamaica | 2.03(1.54,2.57) | 2.3(1.62,3.1) | 0.65(-0.67,1.98) | 0.335 |
| Japan | 6.6(5.88,7.02) | 3.5(2.87,3.89) | -1.97(-2.75,-1.19) | ＜0.001 |
| Jordan | 0.27(0.13,0.52) | 0.27(0.14,0.48) | 0.02(-0.73,0.78) | 0.958 |
| Kazakhstan | 0.05(0.03,0.06) | 1.35(1.01,1.75) | 12.01(10.15,13.9) | ＜0.001 |
| Kenya | 0.2(0.07,1.64) | 0.17(0.05,1.59) | -0.61(-0.7,-0.53) | ＜0.001 |
| Kiribati | 0(0,0.01) | 0(0,0.01) | -0.97(-1.13,-0.81) | ＜0.001 |
| Kuwait | 0.27(0.2,0.34) | 1.02(0.73,1.37) | 3.82(0.78,6.94) | 0.013 |
| Kyrgyzstan | 1.58(1.15,2.28) | 3.04(2.18,4.3) | 1.46(-1.3,4.3) | 0.302 |
| Lao People's Democratic Republic | 2.59(1.26,4.21) | 2.19(1.03,3.45) | -0.54(-0.58,-0.5) | ＜0.001 |
| Latvia | 3.02(2.45,3.64) | 3.19(2.55,3.98) | 0.23(-0.95,1.44) | 0.701 |
| Lebanon | 0.63(0.33,1.12) | 0.3(0.17,0.5) | -2.33(-2.51,-2.14) | ＜0.001 |
| Lesotho | 3.61(1.54,5.92) | 4.41(1.18,7.45) | 0.7(0.36,1.04) | ＜0.001 |
| Liberia | 0.07(0.02,0.15) | 0.04(0.02,0.1) | -1.49(-1.79,-1.19) | ＜0.001 |
| Libya | 0.32(0.15,0.6) | 0.79(0.35,1.45) | 3.01(2.51,3.52) | ＜0.001 |
| Lithuania | 3.72(3.05,4.44) | 4.46(3.58,5.46) | 0.58(-0.38,1.56) | 0.237 |
| Luxembourg | 3.24(2.66,3.9) | 1.5(1.17,1.87) | -2.47(-3.33,-1.61) | ＜0.001 |
| Madagascar | 0.22(0.06,1.72) | 0.13(0.03,1.27) | -1.57(-1.76,-1.39) | ＜0.001 |
| Malawi | 0.29(0.08,2.44) | 0.21(0.05,2.21) | -1.05(-1.18,-0.92) | ＜0.001 |
| Malaysia | 0.61(0.31,1.1) | 0.44(0.23,0.77) | -1(-1.72,-0.28) | 0.007 |
| Maldives | 2.1(1.3,3.36) | 1.29(0.79,1.91) | -1.6(-1.78,-1.42) | ＜0.001 |
| Mali | 0.09(0.03,0.2) | 0.06(0.02,0.13) | -1.32(-1.56,-1.09) | ＜0.001 |
| Malta | 3.12(2.54,3.73) | 1.63(1.25,2.03) | -2.42(-3.08,-1.74) | ＜0.001 |
| Marshall Islands | 0.01(0,0.03) | 0(0,0.01) | -2.44(-2.52,-2.37) | ＜0.001 |
| Mauritania | 0.09(0.03,0.2) | 0.04(0.01,0.1) | -2.32(-2.53,-2.1) | ＜0.001 |
| Mauritius | 0.15(0.11,0.18) | 2.23(1.77,2.76) | 8(-1.96,18.96) | 0.119 |
| Mexico | 15.58(14.43,16.47) | 8.26(7.08,9.39) | -2.06(-2.76,-1.35) | ＜0.001 |
| Micronesia (Federated States of) | 0.01(0,0.03) | 0(0,0.01) | -2.75(-2.8,-2.7) | ＜0.001 |
| Monaco | 0.19(0.1,0.33) | 0.15(0.08,0.26) | -0.74(-0.83,-0.65) | ＜0.001 |
| Mongolia | 1.27(0.64,3.75) | 2.53(1.4,4.06) | 2.12(1.58,2.65) | ＜0.001 |
| Montenegro | 1.03(0.69,1.45) | 1.14(0.72,1.65) | 0.48(0.11,0.86) | 0.012 |
| Morocco | 0.45(0.16,1.15) | 0.83(0.3,1.88) | 2.03(1.79,2.28) | ＜0.001 |
| Mozambique | 0.29(0.07,2.47) | 0.22(0.05,2.31) | -0.76(-0.94,-0.58) | ＜0.001 |
| Myanmar | 2.24(1.07,3.53) | 1.98(0.98,3.11) | -0.4(-0.46,-0.35) | ＜0.001 |
| Namibia | 3.9(1.77,6.7) | 3.22(0.89,5.96) | -0.55(-0.79,-0.32) | ＜0.001 |
| Nauru | 0.01(0,0.02) | 0(0,0.01) | -1.71(-1.78,-1.65) | ＜0.001 |
| Nepal | 6.47(3.32,11.43) | 6.17(3.21,10.82) | -0.13(-0.25,-0.02) | 0.026 |
| Netherlands | 9.5(7.87,11.15) | 4.4(3.45,5.32) | -2.49(-2.9,-2.07) | ＜0.001 |
| New Zealand | 7.52(6.27,8.86) | 3.85(3.01,4.71) | -2.05(-3.68,-0.39) | 0.016 |
| Nicaragua | 4.56(2.84,6.93) | 3.83(2.49,5.7) | -0.59(-1.16,-0.02) | 0.043 |
| Niger | 0.08(0.03,0.18) | 0.05(0.02,0.12) | -1.33(-1.53,-1.14) | ＜0.001 |
| Nigeria | 0.06(0.03,0.11) | 0.04(0.02,0.08) | -1.61(-1.83,-1.39) | ＜0.001 |
| Niue | 0.01(0,0.02) | 0(0,0.01) | -2.86(-2.97,-2.75) | ＜0.001 |
| North Macedonia | 0.55(0.33,0.84) | 0.32(0.17,0.54) | -1.76(-2.11,-1.41) | ＜0.001 |
| Northern Mariana Islands | 0.01(0.01,0.03) | 0.03(0.01,0.06) | 2.18(-0.04,4.44) | 0.054 |
| Norway | 10.31(9.18,11.2) | 3.51(2.93,3.99) | -3.42(-4.83,-1.98) | ＜0.001 |
| Oman | 0.26(0.13,0.5) | 0.28(0.14,0.51) | 0.33(0,0.67) | 0.052 |
| Pakistan | 7.7(4.42,12.18) | 8.65(4.94,13.51) | 0.38(0.32,0.44) | ＜0.001 |
| Palau | 0.03(0.01,0.06) | 0.02(0.01,0.04) | -1.25(-1.55,-0.96) | ＜0.001 |
| Palestine | 0.62(0.32,1.01) | 0.41(0.23,0.66) | -1.4(-1.72,-1.07) | ＜0.001 |
| Panama | 3.05(2.49,3.66) | 2.87(2.08,3.65) | -0.05(-0.65,0.56) | 0.882 |
| Papua New Guinea | 0.01(0,0.02) | 0(0,0.01) | -2.09(-2.22,-1.97) | ＜0.001 |
| Paraguay | 2.03(1.32,3.06) | 3.71(2.2,5.61) | 2.05(1.7,2.4) | ＜0.001 |
| Peru | 5.56(3.31,7.89) | 3.09(1.94,4.62) | -1.86(-2.77,-0.94) | ＜0.001 |
| Philippines | 3.3(1.75,4.23) | 2.43(1.56,2.97) | -0.95(-1.31,-0.59) | ＜0.001 |
| Poland | 6.69(6.24,7.06) | 2.48(2.18,2.74) | -3.34(-4.38,-2.3) | ＜0.001 |
| Portugal | 3.72(3.1,4.37) | 2.31(1.82,2.82) | -1.54(-2.86,-0.2) | 0.024 |
| Puerto Rico | 4.83(4.03,5.64) | 2.35(1.78,2.97) | -2.37(-2.89,-1.84) | ＜0.001 |
| Qatar | 0.34(0.18,0.61) | 0.25(0.11,0.49) | -1.19(-2.22,-0.16) | 0.024 |
| Republic of Korea | 5.91(3.37,9.82) | 1.55(0.94,2.53) | -4.34(-4.66,-4.02) | ＜0.001 |
| Republic of Moldova | 0.73(0.6,0.89) | 1.12(0.9,1.38) | 1.14(0.02,2.27) | 0.046 |
| Romania | 0.02(0.01,0.02) | 0.01(0.01,0.02) | -0.92(-1.96,0.13) | 0.084 |
| Russian Federation | 2.17(2.06,2.25) | 2.75(2.46,3.03) | 0.79(-0.75,2.35) | 0.316 |
| Rwanda | 0.35(0.1,3) | 0.18(0.04,1.89) | -2.11(-2.22,-2) | ＜0.001 |
| Saint Kitts and Nevis | 6.1(4.55,7.71) | 6.42(4.6,8.31) | 0.38(-0.5,1.26) | 0.399 |
| Saint Lucia | 5.71(4.71,6.86) | 4.67(3.63,5.8) | -0.91(-1.38,-0.44) | ＜0.001 |
| Saint Vincent and the Grenadines | 2.38(1.92,2.87) | 1.61(1.2,2.11) | -1.32(-3.35,0.76) | 0.212 |
| Samoa | 0.01(0,0.02) | 0(0,0.01) | -2.59(-2.67,-2.52) | ＜0.001 |
| San Marino | 1.27(0.74,2.04) | 0.54(0.27,0.97) | -2.9(-3.35,-2.44) | ＜0.001 |
| Sao Tome and Principe | 0.06(0.02,0.12) | 0.03(0.01,0.08) | -1.78(-2.07,-1.48) | ＜0.001 |
| Saudi Arabia | 0.37(0.15,0.75) | 0.4(0.21,0.72) | 0.31(-0.02,0.64) | 0.065 |
| Senegal | 0.07(0.02,0.15) | 0.04(0.02,0.1) | -1.38(-1.77,-1) | ＜0.001 |
| Serbia | 2.28(1.4,3.53) | 1.38(0.84,2.14) | -1.6(-1.84,-1.36) | ＜0.001 |
| Seychelles | 0.27(0.17,0.43) | 0.22(0.13,0.35) | -0.87(-1.72,-0.01) | 0.048 |
| Sierra Leone | 0.06(0.02,0.12) | 0.04(0.01,0.09) | -1.38(-1.65,-1.12) | ＜0.001 |
| Singapore | 1.23(0.96,1.52) | 0.35(0.27,0.44) | -3.95(-5.19,-2.69) | ＜0.001 |
| Slovakia | 1.59(1.03,2.26) | 0.99(0.6,1.53) | -1.59(-1.76,-1.41) | ＜0.001 |
| Slovenia | 5.2(4.28,6.22) | 2.5(1.91,3.12) | -2.45(-3.86,-1.02) | 0.001 |
| Solomon Islands | 0.01(0,0.02) | 0(0,0.01) | -1.6(-1.72,-1.48) | ＜0.001 |
| Somalia | 0.43(0.11,3.02) | 0.3(0.07,2.64) | -1.18(-1.29,-1.07) | ＜0.001 |
| South Africa | 5.65(3.84,7.52) | 4.63(3.59,6.01) | -0.66(-1.17,-0.14) | 0.012 |
| South Sudan | 0.25(0.07,1.85) | 0.18(0.05,1.58) | -1.05(-1.16,-0.95) | ＜0.001 |
| Spain | 2.99(2.41,3.66) | 1.67(1.28,2.08) | -1.7(-2.09,-1.31) | ＜0.001 |
| Sri Lanka | 0.28(0.18,0.42) | 0.24(0.13,0.38) | -0.4(-0.77,-0.02) | 0.039 |
| Sudan | 0.47(0.19,1.05) | 0.63(0.3,1.18) | 0.94(0.81,1.06) | ＜0.001 |
| Suriname | 1.18(0.64,2.02) | 0.91(0.47,1.59) | -0.7(-1.35,-0.05) | 0.036 |
| Sweden | 6.3(5.31,7.28) | 3.27(2.55,3.99) | -2.22(-3.27,-1.16) | ＜0.001 |
| Switzerland | 4.65(3.66,5.69) | 2.06(1.52,2.59) | -2.5(-3.35,-1.66) | ＜0.001 |
| Syrian Arab Republic | 0.39(0.21,0.63) | 0.59(0.34,0.88) | 1.31(0.97,1.65) | ＜0.001 |
| Taiwan (Province of China) | 4.27(3.67,4.9) | 2.52(1.99,3.12) | -1.82(-3.49,-0.11) | 0.037 |
| Tajikistan | 0.2(0.1,0.47) | 0.7(0.28,1.2) | 4.19(3.5,4.89) | ＜0.001 |
| Thailand | 1.89(1.03,3.29) | 1.39(0.8,2.31) | -0.99(-1.3,-0.68) | ＜0.001 |
| Timor-Leste | 1.8(0.9,2.92) | 1.92(0.85,3.11) | 0.2(0.14,0.26) | ＜0.001 |
| Togo | 0.06(0.02,0.11) | 0.04(0.01,0.09) | -1.01(-1.26,-0.77) | ＜0.001 |
| Tokelau | 0.01(0,0.03) | 0(0,0.01) | -3.22(-3.4,-3.04) | ＜0.001 |
| Tonga | 0.01(0,0.03) | 0(0,0.01) | -2.13(-2.27,-1.99) | ＜0.001 |
| Trinidad and Tobago | 8.32(6.78,9.89) | 5.01(3.71,6.44) | -1.5(-1.82,-1.17) | ＜0.001 |
| Tunisia | 0.25(0.12,0.46) | 0.41(0.18,0.77) | 1.56(1.29,1.84) | ＜0.001 |
| Turkey | 3.07(1.72,5.14) | 1.77(1.07,2.72) | -1.73(-2.11,-1.35) | ＜0.001 |
| Turkmenistan | 0.01(0.01,0.01) | 0.56(0.4,0.82) | 14.47(10.02,19.1) | ＜0.001 |
| Tuvalu | 0.01(0,0.04) | 0(0,0.01) | -3.01(-3.05,-2.96) | ＜0.001 |
| Uganda | 0.23(0.06,1.69) | 0.17(0.04,1.8) | -0.89(-1.01,-0.77) | ＜0.001 |
| Ukraine | 0.77(0.46,0.98) | 0.76(0.52,1.07) | -0.05(-0.68,0.59) | 0.888 |
| United Arab Emirates | 1.11(0.6,2.01) | 1.04(0.57,1.71) | -0.21(-2.15,1.76) | 0.83 |
| United Kingdom | 11.47(10.57,12.01) | 4.91(4.32,5.29) | -2.97(-4.59,-1.32) | ＜0.001 |
| United Republic of Tanzania | 0.26(0.07,2.14) | 0.16(0.04,1.64) | -1.53(-1.6,-1.46) | ＜0.001 |
| United States of America | 3.53(3.18,3.75) | 2.57(2.19,2.86) | -2.03(-2.49,-1.57) | ＜0.001 |
| United States Virgin Islands | 2.11(1.2,3.46) | 1.13(0.61,1.97) | -1.17(-1.85,-0.49) | 0.001 |
| Uruguay | 3.94(3.26,4.67) | 4.6(3.72,5.51) | 0.47(-0.45,1.41) | 0.319 |
| Uzbekistan | 0.22(0.16,0.45) | 1.73(1.38,2.11) | 6.71(3.73,9.77) | ＜0.001 |
| Vanuatu | 0.01(0,0.02) | 0(0,0.01) | -1.73(-1.93,-1.54) | ＜0.001 |
| Venezuela (Bolivarian Republic of) | 6.84(5.71,8.02) | 5.62(4.05,7.49) | -0.72(-2.3,0.89) | 0.379 |
| Viet Nam | 1.81(0.88,2.93) | 1.8(0.9,2.93) | -0.01(-0.11,0.1) | 0.899 |
| Yemen | 0.46(0.2,0.94) | 0.7(0.33,1.34) | 1.39(1.23,1.55) | ＜0.001 |
| Zambia | 0.27(0.08,2.15) | 0.17(0.04,1.6) | -1.48(-1.66,-1.31) | ＜0.001 |
| Zimbabwe | 2.93(1.33,4.49) | 3.15(1.31,4.9) | 0.34(-0.17,0.85) | 0.198 |
| UI = uncertainty interval. AAPCs = average annual percentage changes. CI = confidence interval. * The P-value was determined by joinpoint regression analysis. | | | | |
|  |  |  |  |  |

**Supplementary Table 4.** The DALYs and AAPCs of rheumatoid arthritis among people aged 60 years and older from 1990 to 2021.

|  | Incidence per 100000 population (95% UI)in 1990 | Incidence per 100000 population (95% UI)in 2021 | AAPCs (95% CI), 1990 to 2021 | P value * |
| --- | --- | --- | --- | --- |
| country |  |  |  |  |
| Afghanistan | 33.2(19.67,55.1) | 53.46(31.5,87.84) | 1.56(1.34,1.78) | ＜0.001 |
| Albania | 75.04(54,101.89) | 75.18(52.33,103.54) | -0.02(-0.26,0.21) | 0.842 |
| Algeria | 20.05(12.54,29.81) | 30.43(19.8,43.79) | 1.36(1.16,1.55) | ＜0.001 |
| American Samoa | 18.73(10.92,29.19) | 22.24(13.13,33.9) | 0.56(0.42,0.7) | ＜0.001 |
| Andorra | 59.39(39.86,83.82) | 59.36(38.99,85.12) | 0(-0.08,0.08) | 0.971 |
| Angola | 40.48(24.45,101.34) | 48.95(30.18,93.04) | 0.62(0.56,0.68) | ＜0.001 |
| Antigua and Barbuda | 83.02(64.01,106.66) | 109.85(84.19,140.67) | 0.95(0.39,1.52) | 0.001 |
| Argentina | 93.51(72.95,117.97) | 113.93(87.38,146.9) | 0.59(0.26,0.93) | 0.001 |
| Armenia | 23.19(13.63,36.19) | 45.23(30.99,63.82) | 2.02(0.17,3.91) | 0.032 |
| Australia | 279.63(222.01,348.79) | 238.01(177.03,311.95) | -0.55(-0.78,-0.32) | ＜0.001 |
| Austria | 186.16(143.99,233.7) | 139.78(101.43,186.46) | -0.94(-1.29,-0.59) | ＜0.001 |
| Azerbaijan | 19.27(10.96,30.51) | 24.41(14.74,36.79) | 0.76(0.63,0.89) | ＜0.001 |
| Bahamas | 94.67(72.51,119.52) | 101.86(77.84,131.36) | 0.3(-0.31,0.93) | 0.333 |
| Bahrain | 42.9(27.82,60.84) | 98.44(70.51,133.55) | 2.69(2.43,2.96) | ＜0.001 |
| Bangladesh | 138.93(85.41,214.94) | 120.85(78.8,179.39) | -0.38(-0.78,0.03) | 0.069 |
| Barbados | 142.97(112.24,179.16) | 173.64(132.37,221.71) | 0.7(0.28,1.13) | 0.001 |
| Belarus | 28.1(17.16,43) | 44.57(28.14,65.11) | 1.49(1.34,1.63) | ＜0.001 |
| Belgium | 161.84(123.34,208.29) | 145.56(105.15,193.11) | -0.29(-0.56,-0.02) | 0.036 |
| Belize | 72.32(54.86,94.31) | 94.33(72.36,120.86) | 0.87(0.5,1.24) | ＜0.001 |
| Benin | 19.01(11.5,29.63) | 23.17(13.59,35.08) | 0.63(0.56,0.69) | ＜0.001 |
| Bermuda | 98.19(74.57,127.47) | 105.86(77.07,141.48) | 0.27(-0.02,0.56) | 0.067 |
| Bhutan | 182.62(112.88,266.87) | 193.07(127.02,275.11) | 0.2(0.15,0.25) | ＜0.001 |
| Bolivia (Plurinational State of) | 192.5(138.66,261.34) | 220.53(157.79,293.17) | 0.44(0.31,0.57) | ＜0.001 |
| Bosnia and Herzegovina | 103.78(75.69,137.92) | 119.92(86.16,163.07) | 0.47(0.36,0.59) | ＜0.001 |
| Botswana | 111.51(72.38,156.75) | 105.71(68.18,147) | -0.2(-0.62,0.21) | 0.332 |
| Brazil | 67.65(54.27,83.4) | 66.21(52.68,82.39) | -0.07(-0.22,0.09) | 0.407 |
| Brunei Darussalam | 232.39(163.37,319.07) | 201.35(142.05,276.43) | -0.51(-0.64,-0.38) | ＜0.001 |
| Bulgaria | 44.86(32.85,60.72) | 48.01(33.01,67.15) | 0.31(0.01,0.61) | 0.044 |
| Burkina Faso | 19.03(11.03,29.02) | 22.11(13.06,34.35) | 0.48(0.36,0.61) | ＜0.001 |
| Burundi | 34.6(20.54,73) | 35.58(21.79,60.79) | 0.09(0.02,0.17) | 0.018 |
| Cabo Verde | 19.26(10.76,30.44) | 27.67(16.31,41.92) | 1.18(1.11,1.26) | ＜0.001 |
| Cambodia | 58.21(35.45,83.29) | 70.28(45.29,97.97) | 0.61(0.57,0.66) | ＜0.001 |
| Cameroon | 22.07(12.89,33.92) | 26.19(15.46,39.83) | 0.57(0.51,0.62) | ＜0.001 |
| Canada | 193.85(153.1,240.7) | 196.94(146.34,258.02) | 0.07(-0.2,0.33) | 0.632 |
| Central African Republic | 44.36(26.99,123.54) | 41.85(25.3,96.84) | -0.19(-0.25,-0.14) | ＜0.001 |
| Chad | 16.7(9.56,25.86) | 18.05(10.73,27.61) | 0.25(0.17,0.32) | ＜0.001 |
| Chile | 193.46(155.91,236.19) | 223.93(167.34,287.87) | 0.5(0.02,0.97) | 0.041 |
| China | 156.44(124.99,197.34) | 153.42(117.63,194.85) | -0.05(-0.26,0.16) | 0.654 |
| Colombia | 142.88(117.32,171.51) | 141.87(108.83,181.13) | -0.13(-0.8,0.54) | 0.701 |
| Comoros | 36.79(22.43,67.09) | 43.24(26.85,72.08) | 0.54(0.48,0.59) | ＜0.001 |
| Congo | 54(33.58,135.36) | 62.09(40.03,111.42) | 0.46(0.39,0.53) | ＜0.001 |
| Cook Islands | 13.99(7.68,22.4) | 19.13(11.18,29.61) | 1.02(0.92,1.12) | ＜0.001 |
| Costa Rica | 172.15(138.16,213.5) | 225.86(173.64,286.35) | 0.91(0.72,1.1) | ＜0.001 |
| Croatia | 124.68(97.85,156.85) | 151.96(111.33,202.32) | 0.67(0.46,0.89) | ＜0.001 |
| Cuba | 93.77(72.12,118.07) | 117.37(89.19,152.7) | 0.7(0.53,0.87) | ＜0.001 |
| Cyprus | 370.47(257.88,520.19) | 274.87(195.15,370.33) | -0.95(-1.13,-0.77) | ＜0.001 |
| Czechia | 69.6(53.46,89.85) | 75.53(54.35,101.64) | 0.29(-0.05,0.63) | 0.099 |
| Democratic People's Republic of Korea | 149.87(107.9,203.29) | 174.58(125.53,234.8) | 0.5(0.44,0.56) | ＜0.001 |
| Democratic Republic of the Congo | 39.44(23.66,99.44) | 44.38(27.49,94.83) | 0.38(0.31,0.45) | ＜0.001 |
| Denmark | 227.67(175.54,288.6) | 263.67(195.72,342.36) | 0.55(0.44,0.65) | ＜0.001 |
| Djibouti | 35.12(21.14,64.5) | 42.6(26.84,68.7) | 0.63(0.57,0.69) | ＜0.001 |
| Dominica | 96.4(68.22,131.62) | 97.62(69.64,132.1) | 0.04(-0.03,0.11) | 0.311 |
| Dominican Republic | 96.27(60.11,135.35) | 79.87(53.84,112.35) | -0.56(-0.82,-0.29) | ＜0.001 |
| Ecuador | 195.01(160.83,233.15) | 212.83(163.34,270.8) | 0.15(-1.1,1.42) | 0.81 |
| Egypt | 27.93(18.6,39.15) | 34.09(22.66,47.38) | 0.67(0.62,0.71) | ＜0.001 |
| El Salvador | 59.47(41.69,82.16) | 74(51.46,102.26) | 0.75(0.72,0.79) | ＜0.001 |
| Equatorial Guinea | 41.98(24.9,118.37) | 68.32(44.77,112.59) | 1.58(1.51,1.65) | ＜0.001 |
| Eritrea | 35.43(20.82,75.39) | 41.2(24.96,74.79) | 0.48(0.38,0.57) | ＜0.001 |
| Estonia | 183.39(145.07,228.51) | 205.19(161.15,256.81) | 0.38(0.05,0.71) | 0.024 |
| Eswatini | 149.2(87.22,210.75) | 142(77.46,212.67) | -0.14(-0.29,0.01) | 0.061 |
| Ethiopia | 38.79(25.26,75.87) | 33.52(21.83,53.91) | -0.47(-0.51,-0.43) | ＜0.001 |
| Fiji | 11.52(6.52,18) | 13.35(7.52,21.25) | 0.48(0.41,0.55) | ＜0.001 |
| Finland | 407.54(331.08,497.26) | 324.37(243.08,419.4) | -0.75(-0.91,-0.59) | ＜0.001 |
| France | 152.23(118.77,193.47) | 134.53(96.47,180.78) | -0.43(-0.65,-0.2) | ＜0.001 |
| Gabon | 56.16(35.08,121.17) | 72.4(46.25,119.64) | 0.82(0.77,0.87) | ＜0.001 |
| Gambia | 19.59(11.39,30.01) | 24.15(14.14,36.66) | 0.67(0.61,0.74) | ＜0.001 |
| Georgia | 24.25(13.66,37.67) | 33.11(21.74,48.27) | 0.74(-0.91,2.41) | 0.381 |
| Germany | 142.03(108.8,181.87) | 123.15(89.87,163.91) | -0.44(-0.69,-0.18) | 0.001 |
| Ghana | 17.72(10.18,27.66) | 29.7(16.82,47.63) | 1.7(1.47,1.93) | ＜0.001 |
| Greece | 86.4(61.54,115.29) | 98.14(69.09,132.61) | 0.4(0.22,0.59) | ＜0.001 |
| Greenland | 112.64(80.31,157.46) | 136.34(96.49,186.31) | 0.72(0.55,0.88) | ＜0.001 |
| Grenada | 45.65(32.76,61.86) | 63.9(46.77,84.66) | 1.16(0.99,1.33) | ＜0.001 |
| Guam | 16.97(9.83,26.69) | 22.5(12.94,34.03) | 0.93(0.75,1.11) | ＜0.001 |
| Guatemala | 103.7(86.2,125.59) | 136.92(109.07,170.76) | 0.89(0,1.78) | 0.049 |
| Guinea | 18.05(10.57,27.94) | 20.55(12.23,31.92) | 0.45(0.4,0.49) | ＜0.001 |
| Guinea-Bissau | 18.26(10.57,28.33) | 21.68(12.51,33.41) | 0.55(0.5,0.6) | ＜0.001 |
| Guyana | 22.2(13.6,33.72) | 46.75(33.99,62.58) | 2.39(1.67,3.12) | ＜0.001 |
| Haiti | 104.17(61.74,172.52) | 93.65(56.52,153.72) | -0.34(-0.41,-0.26) | ＜0.001 |
| Honduras | 225.08(149.87,341.85) | 316.63(228.07,423.51) | 1.11(0.89,1.33) | ＜0.001 |
| Hungary | 145.7(119.24,178.73) | 128.83(97.19,166.78) | -0.42(-0.54,-0.3) | ＜0.001 |
| Iceland | 157.1(120.48,201.96) | 144.01(103.81,192.17) | -0.33(-0.47,-0.2) | ＜0.001 |
| India | 183.94(135.72,239.83) | 208.87(161.62,270.6) | 0.45(0.22,0.68) | ＜0.001 |
| Indonesia | 25.79(16.66,35.26) | 33.62(21.3,45.16) | 0.87(0.82,0.92) | ＜0.001 |
| Iran (Islamic Republic of) | 23.21(16.54,31.32) | 30.51(22.36,40.93) | 0.88(0.79,0.97) | ＜0.001 |
| Iraq | 23.59(15.18,34.12) | 30.96(20.15,44.96) | 0.89(0.81,0.97) | ＜0.001 |
| Ireland | 423.49(338.71,520.08) | 375.51(277.74,487.64) | -0.38(-0.77,0.01) | 0.057 |
| Israel | 91.32(67.79,121.99) | 102.79(72.32,139.18) | 0.41(0.12,0.7) | 0.006 |
| Italy | 168.4(129.36,215.3) | 153.05(115.35,198.99) | -0.33(-0.68,0.03) | 0.076 |
| Jamaica | 62.56(46.93,81.36) | 79.31(59.72,103.15) | 0.85(0.27,1.43) | 0.004 |
| Japan | 282.33(226.38,350.2) | 192.57(147,247.84) | -1.18(-1.48,-0.88) | ＜0.001 |
| Jordan | 23.3(15.08,34.84) | 32.64(21.17,46.81) | 1.12(1.01,1.23) | ＜0.001 |
| Kazakhstan | 22.9(13.19,35.84) | 50.38(36.28,68.01) | 2.68(1.36,4.01) | ＜0.001 |
| Kenya | 37.8(25.59,61.62) | 40.85(27.65,65.89) | 0.25(0.22,0.28) | ＜0.001 |
| Kiribati | 10.93(6.43,16.92) | 12.75(7.35,19.53) | 0.5(0.41,0.59) | ＜0.001 |
| Kuwait | 36.36(24.2,51.64) | 61.98(44.33,83.43) | 1.7(0.68,2.74) | 0.001 |
| Kyrgyzstan | 106.31(75.53,143.6) | 172.45(127.56,230.1) | 1.36(0.14,2.59) | 0.029 |
| Lao People's Democratic Republic | 58.56(35.52,86.27) | 60.13(37.93,84.86) | 0.08(0.04,0.12) | ＜0.001 |
| Latvia | 135.17(105.58,167.02) | 152.68(119.65,193.82) | 0.4(-0.1,0.91) | 0.116 |
| Lebanon | 36.24(24.24,50.35) | 42.02(26.81,61.07) | 0.51(0.39,0.63) | ＜0.001 |
| Lesotho | 108.28(71.05,155.31) | 130.08(72.62,187.02) | 0.63(0.44,0.82) | ＜0.001 |
| Liberia | 18.46(11.09,28.17) | 23.22(13.63,36) | 0.75(0.68,0.82) | ＜0.001 |
| Libya | 23.28(14.9,34.17) | 38.62(25.35,55.41) | 1.67(1.5,1.84) | ＜0.001 |
| Lithuania | 159.07(126.61,196.26) | 193.72(152.6,238.36) | 0.65(0.21,1.09) | 0.004 |
| Luxembourg | 138.9(106.85,176.5) | 123.13(88.6,165.98) | -0.43(-0.67,-0.19) | ＜0.001 |
| Madagascar | 29.26(17.45,55.03) | 33.52(20.69,55.49) | 0.43(0.39,0.47) | ＜0.001 |
| Malawi | 36.49(22.72,72.6) | 43.77(27.54,77.59) | 0.6(0.52,0.67) | ＜0.001 |
| Malaysia | 24.16(15.75,36) | 25.45(16.27,36.91) | 0.12(-0.08,0.33) | 0.236 |
| Maldives | 60.95(43.11,85.37) | 62.63(42.96,86.62) | 0.02(-0.14,0.17) | 0.846 |
| Mali | 17.04(10.01,26.05) | 19.79(11.59,30.18) | 0.48(0.41,0.56) | ＜0.001 |
| Malta | 129.57(99.39,162.98) | 117.91(85.46,158.58) | -0.43(-0.67,-0.18) | 0.001 |
| Marshall Islands | 15.41(8.72,24.25) | 19.36(11.41,29.99) | 0.73(0.67,0.78) | ＜0.001 |
| Mauritania | 21.62(12.53,33.15) | 29.66(17.84,44.41) | 1.05(1.02,1.08) | ＜0.001 |
| Mauritius | 27.69(17.09,41.01) | 74.15(57.54,94.35) | 3.12(1.26,5.01) | 0.001 |
| Mexico | 380.51(326,443.78) | 305.11(249.11,372.48) | -0.73(-1.08,-0.37) | ＜0.001 |
| Micronesia (Federated States of) | 16.23(9.47,25.07) | 23.23(13.69,35.44) | 1.16(1.08,1.23) | ＜0.001 |
| Monaco | 53.53(34.28,78.61) | 56.48(36.21,83.15) | 0.18(0.12,0.24) | ＜0.001 |
| Mongolia | 47.64(31.16,87.5) | 82.35(56.19,113.47) | 1.72(1.45,2) | ＜0.001 |
| Montenegro | 64.85(46.28,87.96) | 76.47(54.3,104.18) | 0.58(0.43,0.73) | ＜0.001 |
| Morocco | 23.38(14.16,37.76) | 37.4(22.07,59.25) | 1.54(1.43,1.65) | ＜0.001 |
| Mozambique | 34.12(20.31,69.78) | 40.35(25.27,73.43) | 0.54(0.47,0.61) | ＜0.001 |
| Myanmar | 53.27(32.68,75.98) | 59.07(38.81,82.52) | 0.33(0.27,0.38) | ＜0.001 |
| Namibia | 110.3(70.49,160.57) | 111.48(68.97,164.3) | 0.04(-0.02,0.11) | 0.196 |
| Nauru | 17.65(10.24,27.21) | 24.37(14.74,37.54) | 1.05(0.96,1.14) | ＜0.001 |
| Nepal | 153.19(99.57,233.61) | 177.37(119.74,255.33) | 0.49(0.42,0.55) | ＜0.001 |
| Netherlands | 359.4(285.25,443.2) | 263.53(197.02,343.19) | -0.99(-1.23,-0.76) | ＜0.001 |
| New Zealand | 355.08(276.08,448.26) | 268.68(194.81,356.48) | -0.91(-1.27,-0.54) | ＜0.001 |
| Nicaragua | 136.02(100.37,183) | 159.29(118.73,207.15) | 0.51(0.23,0.79) | ＜0.001 |
| Niger | 16.39(9.63,26.05) | 18.74(10.91,28.78) | 0.45(0.39,0.51) | ＜0.001 |
| Nigeria | 17.89(12.15,25.38) | 20.62(13.85,29.16) | 0.47(0.41,0.54) | ＜0.001 |
| Niue | 17.35(10.04,27.2) | 22.89(13.35,34.91) | 0.91(0.85,0.97) | ＜0.001 |
| North Macedonia | 38.19(26,55.15) | 46.34(30.85,67.52) | 0.63(0.55,0.71) | ＜0.001 |
| Northern Mariana Islands | 21.23(12.03,32.99) | 24.52(14.6,37.62) | 0.48(0.26,0.7) | ＜0.001 |
| Norway | 425.56(340.56,527.7) | 259.61(191.92,343.49) | -1.53(-1.79,-1.26) | ＜0.001 |
| Oman | 19.8(12.71,29.53) | 30.66(19.8,44.21) | 1.43(1.26,1.6) | ＜0.001 |
| Pakistan | 181.25(123.71,253.02) | 191.61(126.08,272.44) | 0.19(0.14,0.25) | ＜0.001 |
| Palau | 20.27(12.02,30.83) | 25.69(15.39,37.88) | 0.78(0.7,0.86) | ＜0.001 |
| Palestine | 31.21(20.56,44.62) | 35.82(24,51.9) | 0.44(0.32,0.56) | ＜0.001 |
| Panama | 98.97(78.67,123.86) | 112.81(84.01,144.85) | 0.49(0.31,0.67) | ＜0.001 |
| Papua New Guinea | 11.66(7.05,17.71) | 13.07(7.38,20.49) | 0.37(0.3,0.44) | ＜0.001 |
| Paraguay | 86.21(62.79,117.25) | 156.77(113.47,207.46) | 1.95(1.81,2.1) | ＜0.001 |
| Peru | 147.34(103.08,195.82) | 182.58(130.68,244.66) | 0.72(0.41,1.02) | ＜0.001 |
| Philippines | 80.97(55.66,102.74) | 69.51(52.37,86.61) | -0.45(-0.67,-0.23) | ＜0.001 |
| Poland | 234.8(202.53,272.9) | 137.1(108.81,171.03) | -1.81(-2.23,-1.38) | ＜0.001 |
| Portugal | 135.04(106.22,167.78) | 143.23(105.48,187.24) | 0.2(-0.18,0.58) | 0.299 |
| Puerto Rico | 106.01(81.79,134.34) | 113.46(82.31,150.15) | 0.2(0.04,0.37) | 0.017 |
| Qatar | 26.9(17.28,38.78) | 42.19(27.2,60.78) | 1.5(1.22,1.79) | ＜0.001 |
| Republic of Korea | 159.34(113.51,222.93) | 129.37(91.2,173.62) | -0.69(-0.78,-0.6) | ＜0.001 |
| Republic of Moldova | 41.56(30.01,56.56) | 62.96(46.81,83.03) | 1.31(0.77,1.85) | ＜0.001 |
| Romania | 27.89(16.8,42.57) | 44.95(28.08,67.67) | 1.55(1.48,1.61) | ＜0.001 |
| Russian Federation | 95(79.66,113.83) | 108(91.08,129.35) | 0.42(-0.12,0.97) | 0.126 |
| Rwanda | 38.07(23.12,82.98) | 48.02(29.95,79.42) | 0.75(0.62,0.89) | ＜0.001 |
| Saint Kitts and Nevis | 130.5(101.24,162.71) | 155.33(118.58,195.46) | 0.6(0.36,0.84) | ＜0.001 |
| Saint Lucia | 114.8(91.77,141.57) | 116.74(89.94,147.72) | -0.13(-0.39,0.13) | 0.327 |
| Saint Vincent and the Grenadines | 58.15(44.38,74.78) | 52.7(38.03,70.72) | -0.34(-1.09,0.41) | 0.368 |
| Samoa | 17.38(10.14,26.8) | 22.4(13.33,33.71) | 0.83(0.77,0.89) | ＜0.001 |
| San Marino | 69.66(48.08,97.36) | 60.07(40.15,86.18) | -0.51(-0.64,-0.38) | ＜0.001 |
| Sao Tome and Principe | 21.11(12.4,32.62) | 27.99(16.53,43.17) | 0.91(0.81,1.02) | ＜0.001 |
| Saudi Arabia | 21.25(13.56,31.56) | 31.34(20.54,44.81) | 1.26(1.18,1.33) | ＜0.001 |
| Senegal | 19.91(11.65,30.3) | 24.53(14.56,37.85) | 0.7(0.64,0.75) | ＜0.001 |
| Serbia | 83.68(60.37,113.51) | 91.53(65.66,122.53) | 0.32(0.21,0.44) | ＜0.001 |
| Seychelles | 20.23(12.98,29.94) | 24.03(14.64,35.69) | 0.53(0.34,0.72) | ＜0.001 |
| Sierra Leone | 18.29(10.73,28.39) | 21.11(12.32,32.51) | 0.48(0.41,0.55) | ＜0.001 |
| Singapore | 62.57(46.45,83.79) | 60.31(40.03,86.6) | -0.1(-0.37,0.17) | 0.459 |
| Slovakia | 61.98(44.99,82.78) | 64.69(46.23,88.45) | 0.13(0,0.26) | 0.054 |
| Slovenia | 201.42(162.39,247.51) | 191.9(141.15,253.8) | -0.26(-0.64,0.11) | 0.169 |
| Solomon Islands | 13.77(8.07,21.54) | 18.59(10.89,28.09) | 0.97(0.92,1.02) | ＜0.001 |
| Somalia | 39.18(23.77,82.14) | 38.12(22.99,78.59) | -0.08(-0.1,-0.07) | ＜0.001 |
| South Africa | 184.61(141.07,232.81) | 160.53(126.41,201.39) | -0.46(-0.73,-0.19) | 0.001 |
| South Sudan | 31.42(19.26,59.19) | 34.76(21.43,59.4) | 0.32(0.25,0.4) | ＜0.001 |
| Spain | 136.87(106.68,172.17) | 132.54(96.01,177.69) | -0.09(-0.22,0.04) | 0.179 |
| Sri Lanka | 18.13(12.03,26.22) | 22.63(13.84,33.83) | 0.74(0.47,1.01) | ＜0.001 |
| Sudan | 21.21(13,33.56) | 29.99(20,43.69) | 1.13(1.05,1.22) | ＜0.001 |
| Suriname | 38.61(25.35,56.69) | 39.76(26.55,58.33) | 0.14(-0.11,0.39) | 0.266 |
| Sweden | 296.58(231.15,375.81) | 214.68(157.85,284.11) | -1.1(-1.47,-0.73) | ＜0.001 |
| Switzerland | 215.42(164.79,276.58) | 179.78(129.08,240.72) | -0.59(-0.76,-0.42) | ＜0.001 |
| Syrian Arab Republic | 23.32(15.61,33.1) | 35.26(23.89,48.85) | 1.36(1.27,1.44) | ＜0.001 |
| Taiwan (Province of China) | 143.92(114.71,178.06) | 121.89(92.96,157.05) | -0.6(-0.95,-0.26) | 0.001 |
| Tajikistan | 39.8(25.02,58.92) | 59.01(39.57,83.08) | 1.27(1.12,1.43) | ＜0.001 |
| Thailand | 58.3(39.51,86.92) | 60.96(42.44,85.54) | 0.15(0.01,0.29) | 0.04 |
| Timor-Leste | 44.46(27.84,64.63) | 54.4(34.13,77.77) | 0.66(0.6,0.71) | ＜0.001 |
| Togo | 19.52(11.5,30.3) | 24.32(14.23,37.31) | 0.72(0.66,0.78) | ＜0.001 |
| Tokelau | 14.56(8.44,22.49) | 20.95(12.44,31.71) | 1.19(1.07,1.3) | ＜0.001 |
| Tonga | 16.21(9.35,25.31) | 20.47(11.87,31.59) | 0.76(0.67,0.85) | ＜0.001 |
| Trinidad and Tobago | 169.83(139.65,205.07) | 150.07(116.25,189.01) | -0.32(-0.5,-0.14) | ＜0.001 |
| Tunisia | 20.88(13.28,30.31) | 32.02(21.1,46.3) | 1.39(1.31,1.47) | ＜0.001 |
| Turkey | 94.74(66.07,133.31) | 99.51(71.06,132.96) | 0.16(0.06,0.26) | 0.002 |
| Turkmenistan | 18.78(10.55,29.78) | 33.13(22.77,46.84) | 1.91(1.57,2.26) | ＜0.001 |
| Tuvalu | 14.29(8.23,22.25) | 19.71(11.38,29.93) | 1.07(0.97,1.16) | ＜0.001 |
| Uganda | 34.31(20.91,61.07) | 42.39(25.65,71.07) | 0.7(0.61,0.79) | ＜0.001 |
| Ukraine | 40.31(28.51,53.91) | 46.07(32.99,62.3) | 0.41(0.04,0.78) | 0.029 |
| United Arab Emirates | 43.5(29.38,63.4) | 43.5(28.55,62.46) | -0.01(-0.67,0.65) | 0.973 |
| United Kingdom | 390.13(326.38,465.37) | 286.16(220.26,365.18) | -1.09(-1.64,-0.54) | ＜0.001 |
| United Republic of Tanzania | 36.94(22.19,68.91) | 44.02(27.57,70.69) | 0.56(0.5,0.62) | ＜0.001 |
| United States of America | 181.85(144.6,225.88) | 175.94(134.14,224.61) | -0.23(-0.36,-0.09) | 0.001 |
| United States Virgin Islands | 63.25(43.42,89.57) | 59.37(39.57,86.28) | -0.13(-0.34,0.09) | 0.247 |
| Uruguay | 127.27(101.86,157.53) | 161.61(128.33,201.44) | 0.74(0.51,0.96) | ＜0.001 |
| Uzbekistan | 68.79(44.82,99.87) | 106.37(77.55,142.74) | 1.26(0.42,2.11) | 0.003 |
| Vanuatu | 13.77(7.92,21.34) | 17.18(10.1,26.58) | 0.73(0.67,0.79) | ＜0.001 |
| Venezuela (Bolivarian Republic of) | 198.53(162.85,239.25) | 214.15(166.03,270.88) | 0.15(-0.5,0.81) | 0.649 |
| Viet Nam | 54.9(36.07,77.8) | 77.7(52.81,108.51) | 1.13(1.09,1.18) | ＜0.001 |
| Yemen | 20.59(13.24,31.9) | 28.86(18.82,42.91) | 1.12(1.05,1.19) | ＜0.001 |
| Zambia | 32.64(19.78,63.44) | 40.21(24.91,67.26) | 0.68(0.62,0.74) | ＜0.001 |
| Zimbabwe | 78.95(49.5,109.45) | 84.64(51.28,117.3) | 0.28(0.05,0.51) | 0.019 |
| UI = uncertainty interval. AAPCs = average annual percentage changes. CI = confidence interval. * The P-value was determined by joinpoint regression analysis. | | | | |
|  |  |  |  |  |
